# Submicromolar imaging of intrinsic chromophores by two-photon photothermal microscopy captures mitochondrial response to chemotherapy

**DOI:** 10.1126/sciadv.aee7678

**Published:** 2026-07-10

**Authors:** Nathaniel Hai, Chinmayee Vallabh Prabhu Dessai, Dingcheng Sun, Jianpeng Ao, Pin-Tian Lyu, Yifan Zhu, Ji-Xin Cheng

**Affiliations:** ^1^ Department of Electrical and Computer Engineering, Boston University, Boston, MA 02215, USA.; ^2^ Photonics Center, Boston University, Boston, MA 02215, USA.; ^3^ Department of Biomedical Engineering, Boston University, Boston, MA 02215, USA.

## Abstract

Intracellular chromophores {e.g., NADH [reduced form of nicotinamide adenine dinucleotide (oxidized form)] and FAD (flavin adenine dinucleotide)} play a central role in regulation of cellular metabolism. Although autofluorescence has been extensively used for label-free mapping of chromophores inside a cell, its sensitivity and molecular specificity are constrained by the low quantum yield and the fluorescence spectral overlap. Here, we address these challenges by using a photothermal approach to measure the optical absorption of chromophores rather than its autofluorescence. Our two-photon photothermal (2PPT) microscope exploits localized thermal transients generated through two-photon absorption, enabling detection of chromophore-specific signatures beyond the reach of autofluorescence. We demonstrate submicromolar limits of detection for the metabolic coenzymes NADH and FAD of 0.87 and 0.99 μM, respectively. Such high sensitivity enables differentiating the influence of mitochondrial shapes on metabolism. 2PPT can identify the biomolecular source of contrast from cellular mitochondria in a label-free manner on the basis of spectroscopy. 2PPT microscopy is used to study metabolic alterations of mitochondria in cancer under chemotherapy at the single-organelle level.

## Teaser

Label-free photothermal imaging reveals real-time mitochondrial stress and metabolic shifts at the single-organelle level.

## INTRODUCTION

Label-free, high-resolution imaging has been the foundation of sensing metabolism-related intrinsic chromophores in their native environment with minimal perturbation to their function (*1*, *2*). Building on early works showing the applicability of intrinsic fluorescence (i.e., autofluorescence) to serve as a cancer biomarker (*3*, *4*), the field has rapidly accelerated with subsequent studies on its microstructural and functional mapping (*5*–*8*). Consensus guidelines aimed to standardize data collection across researchers in the field of label-free metabolic imaging were recently published (*9*). Meanwhile, by using multiphoton excitation for better spatial confinement and spectral selectivity (*10*), the optical redox ratio derived from autofluorescence of the reduced form of NAD^+^ [nicotinamide adenine dinucleotide (oxidized form)] (NADH) and flavin adenine dinucleotide (FAD) was shown to correlate with biochemical-based assays that quantify the cellular phosphorylation activity and redox state (*11*). Harnessing fluorescence lifetime microscopy together with the emission intensity to measure the collective optical metabolic index has been identified as a more robust indicator of metabolic response to drug action at a cellular level, owing to the distinct decay times of bound and unbound states of the biomolecule (*12*). Noninvasive quantification of metabolic activity based on multiparametric imaging further identified alterations in specific metabolic pathways and characterized the response of a single cell to them (*13*, *14*). Adopting intrinsic chromophore fluorescence as a contrast agent, fluorescence lifetime microscopy techniques were applied for in vivo characterization of biological tissues (*15*). Since then, intravital imaging modalities were carefully designed to detect the metabolic activity of cells within the tumor microenvironment, which potentially enable in vivo redox state mapping (*7*, *16*–*19*). More recently, studies on patient-extracted cellular organoids and tissues have emerged (*20*–*22*). Collectively, these works highlight the growing interest in such label-free noninvasive imaging approaches to study subcellular metabolic activity and its potential to serve as a diagnosis and therapeutic biomarker.

While the abovementioned autofluorescence-based modalities have been widely used for sensing metabolism-relevant biomolecules in tens of micromolars, they are characterized by inherently weak signal levels (*23*) that are largely influenced by the properties of surrounding media like temperature, pH, and binding partners (*24*). This native fluorescence originates from the radiative decay of the molecule after absorption of excitation light, which is a less probable de-excitation pathway compared to nonradiative pathways (*25*) with a modest quantum yield of 2 to 10% for metabolic coenzymes such as NADH and FAD (*24*, *26*). Several research groups have addressed the low-efficiency issue by creative designs of nanoprobes introduced into the medium of interest and detected by optical absorption or scattering mechanisms (*27*–*29*), achieving an impressive limit of detection (LOD) in the submicromolar regions (*30*). However, the injection of exogenous probes can modify the natural biomolecular behaviors of the metabolism reporters, rendering these approaches ill-posed for highly sensitive detection of the metabolic coenzymes in biomedically relevant scenarios.

Targeting the generated heat during light absorption by the target chromophores has the potential to alleviate the abovementioned challenges. Upon absorption, the chromophore enters an excited state, from which relaxation back to the ground state often occurs through radiative decay (fluorescence emission) or nonradiative decay (e.g., internal conversion and vibrational relaxation) that contributes to heat. In low-quantum-efficiency biomolecules, energy transfer mechanisms are the second-most dominant after pathways that generate local heat (*31*). This implies that efficient sensing of these biomolecules could, in principle, be done by sensing the photothermal effect with extreme signal boost between 10 and 20 times compared with autofluorescence detection. Previous efforts to optically sense the generated heat after photoabsorption have reached single chromophore sensitivity in a synthetic DNA construct (*32*). Furthermore, photothermal imaging within biocompartments have achieved label-free detection of specific chromophores including hemoglobin and nanoparticles (*33*–*35*). Notably, photothermal imaging modalities that rely on multiphoton excitation benefit from several advantages owing to the integration of nonlinear excitation with subsequent photothermal sensing and have shown the capacity to sense cytochrome molecules in kidney cells (*36*).

In the present study, we investigate two-photon photothermal detection (2PPT) of metabolic coenzymes on the basis of a comparison with standard autofluorescence approaches. We test the potential of imaging the photothermally excited biomolecules to deliver higher sensitivity and specificity given fixed experimental conditions. Specifically, 2PPT uses highly efficient heat generation by the intrinsic chromophores compared to their fluorescence signal generation, delivering up to 20 times better LOD of these biomedically relevant coenzymes in a low-concentration environment. Concurrently, 2PPT is shown to differentiate the influence of energetic perturbations in various mitochondrial morphologies, which suggests how conformational changes might be associated with the organelle’s metabolic activity. In addition, our approach delineates modifications in the metabolic activity of live cells and tumor spheroids after targeted energetic perturbations and after chemotherapy drug treatment, respectively.

## RESULTS

### 2PPT microscope

**Fig. 1.**
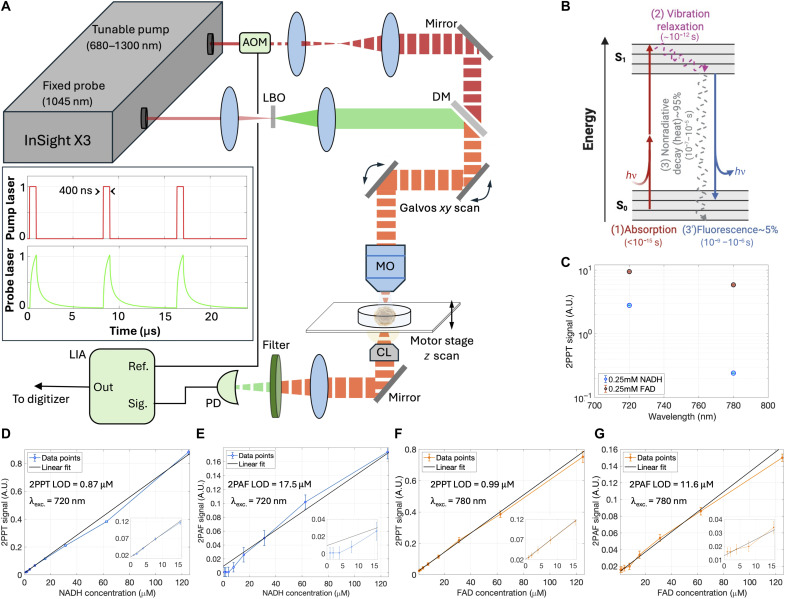
2PPT microscopy working principle and LOD. (**A**) 2PPT microscope benchtop implementation. The inset shows the time trace of pump laser (red) and probe laser (green). LBO, lithium triborate crystal; DM, dichroic mirror; MO, microscope objective; CL, condenser lens; PD, photodetector; LIA, lock-in amplifier. (**B**) Electronic and vibration energy band diagram illustrating the three-step process of signal generation in 2PPT and 2PAF along with characteristic timescales [created in BioRender; N. Hai (2026), https://biorender.com/2zof5pw]. (**C**) 2PPT signal of NADH and FAD sterile samples for estimating their action cross section. A.U., arbitrary units. (**D** and **E**) Plots of 2PPT and 2PAF signals as a function of NADH concentration and the linear fit from which the LOD is calculated. The excitation wavelength is 720 nm. (**F** and **G**) Plots of 2PPT and 2PAF signals as a function of FAD concentration and the linear fit from which the LOD is calculated. The excitation wavelength is 780 nm. Insets of (D) to (G) show data points and linear fit in a low concentration range.

Figure 1A illustrates the 2PPT microscope, which uses a pump beam (red) for excitation and a probe beam (green) for label-free imaging of photothermally generated heat by the target biomolecules in the sample. Our implementation uses a dual-port solid-state ultrafast laser oscillator as the light source for both beams, whereas the tunable near-infrared (NIR) port is used for excitation and combined with the frequency-doubled output of the fixed 1045-nm port for heat sensing with a visible wavelength (522.5 nm). The generation of 2PPT signal can be described as a three-step process and is illustrated in Fig. 1B. After light absorption by the target biomolecule, relatively fast (approximately picoseconds) vibrational relaxation within the excited electronic level occurs, after which the molecule can undergo a slower decay (nano- to microseconds) to a high vibrational state within the ground state and emit a photon (*25*). This process has a low quantum yield of 8 to 10% for FAD and even a lower efficiency of 2 to 4% for NADH (*24*, *26*). However, the more probable event for the electronically excited molecule is to undergo slower thermal relaxation (tenths to tens of microseconds) after the decay back to the ground state without emitting any photon. This process generates locally distributed transient heat that changes the local refractive index (*37*, *38*). Thus, the expression of a specific biomolecule, namely a chromophore, can be reliably measured by optically sensing the change in local refractive index with high sensitivity. To put this hypothesis to test, we use the described pump-probe configuration to sense the locally generated heat within sterile solutions of metabolic coenzymes, namely NADH and FAD, after two-photon (2P) absorption. As illustrated in Fig. 1A, after the combination of the two beams via the dichroic mirror, they are relayed toward the sample plane after passing through a pair of galvanometric mirrors to laser-scan the two beams at the back focal plane of a 60×, 1.2–numerical aperture (NA) water immersion microscope objective (MO). After interacting with the sample, the two beams are collected by a 1.4-NA oil immersion condenser lens (CL) equipped with a variable aperture. After that, the excitation beam is blocked by the chromatic filter, and only the probe beam makes its way toward the single-pixel photodetector connected to a digitization system based on lock-in amplifier (LIA) detection (see Materials and Methods for further details). As mentioned above, the timescale of the chromophore’s thermal relaxation is in the range of 0.1 to 10 μs, which also sets the timescale for transient heat generation that serves as the contrast mechanism in 2PPT microscopy. Thus, to optimize the signal collection, we used an external acousto-optic modulator (AOM) to control the heat generation process. AOM modulation was set to *f* = 125 kHz, which mitigates the 1/*f* laser noise dominant at a low frequency (<100 kHz) while satisfying the requirement for photothermal signal detection (100 kHz < *f* < 10,000 kHz). Decreasing the modulation frequency beyond that point shows a minimal amplitude increase in signal and a more prominent noise increase, which overall reduces the signal-to-noise ratio. Inset plots in Fig. 1A show the signal time traces of pump beam (red plot) and the result in waveform of the probe beam (green plot). One can observe that the initially unmodulated probe beam takes the shape of rapid local heating followed by gradual heat dissipation exactly at the pump beam modulation frequency, as expected by the heat propagation equation (*38*). Notably, the photothermal signal between consecutive pulse trains decays back to zero, which implies the complete dissipation of accumulated heat from the affected area that is important for optimal signal extraction. The modulation parameters for our experiments, e.g., the duty cycle, modulation frequency, and decay time, were verified on the basis of simulation of the heat generation process of NADH solution (see Materials and Methods and fig. S1). An image is formed by two-dimensional (2D) scan of the pump and probe beams together via the galvo mirrors.

**Fig. 2.**
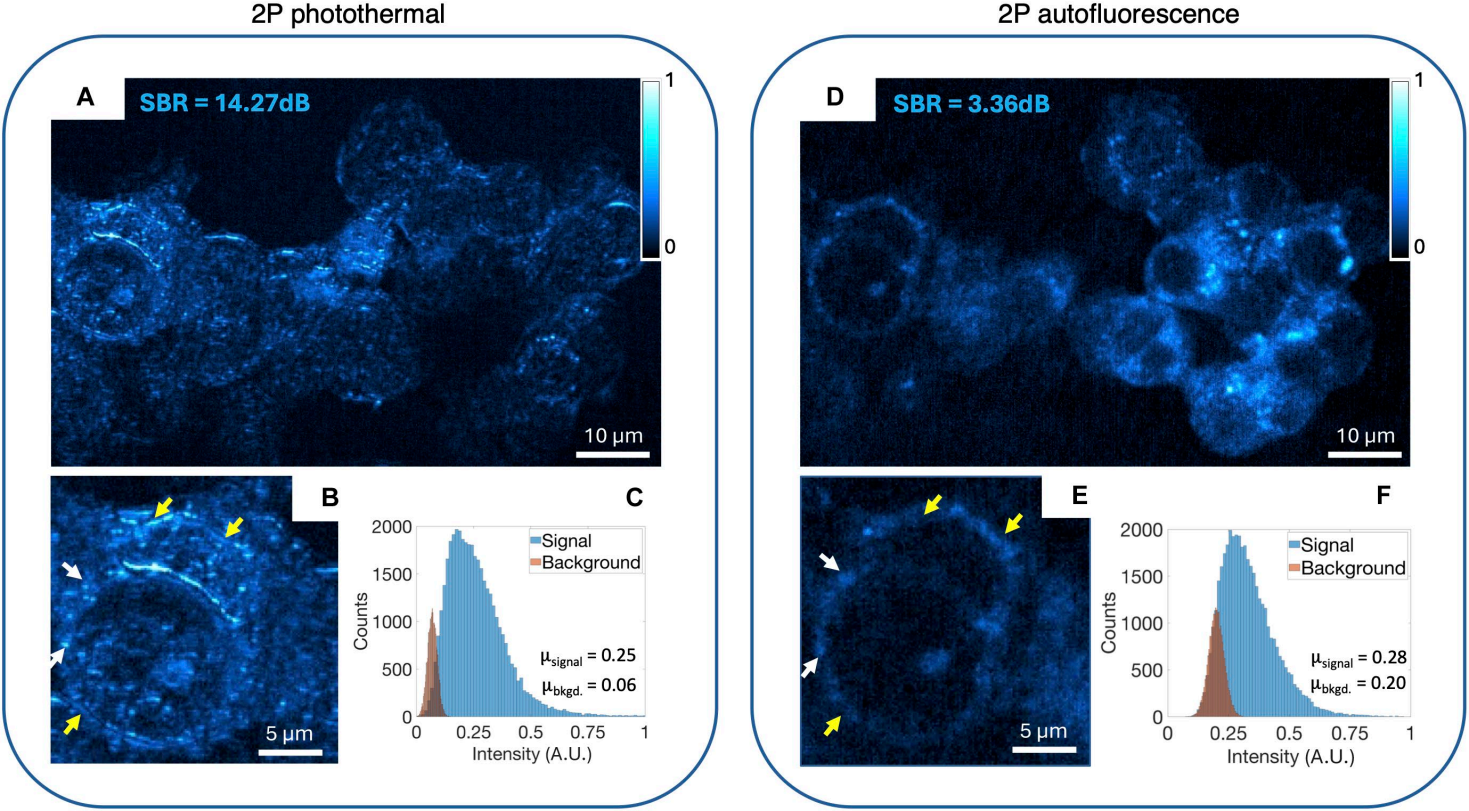
Comparison between 2PPT and 2PAF for imaging mitochondrial NADH in cancerous cells. (**A** and **D**) Monolayer of fresh, energy-producing SK-OV-3 cells obtained with 2PPT and 2PAF, respectively. (**B** and **E**) Single-cell image showing tubular mitochondria (yellow arrows) and blob/oval mitochondria (white arrows) obtained with 2PPT and 2PAF, respectively. (**C** and **F**) Intensity histogram of images obtained with 2PPT and 2PAF, respectively, that shows the signal (blue bins) and background (orange bins) along with the mean values denoted by μ_signal_ and μ_bkgd._.

After we validated that 2P absorption occurs in the sample, both by inherent optical sectioning and by power law scaling (fig. S2) (*10*, *39*), we tested the 2PPT sensing performance for metabolic coenzymes. Figure 1C illustrates the 2PPT signal in the case of 0.25 mM NADH and FAD solutions when using pump excitation at 720 and 780 nm. Our results closely match the 2P action cross sections for the two biomolecules (*23*, *39*), emphasizing that signal generation in both methods is equivalent up to a factor that favors 2PPT (supplementary text). We further tested the sensitivity of 2PPT in detecting the coenzymes and compared it with that of conventional 2P autofluorescence (2PAF). Figure 1 (D and E) plots the observed signal for different concentrations of NADH solutions obtained with 2PPT and 2PAF, respectively, along with a linear fit from which the LOD for each method can be calculated on the basis of the residual standard deviation and the slope (see Materials and Methods and supplementary text) (*40*). The 2PPT data points show lower residuals relative to the linear fit (Fig. 1D) across the measured NADH concentrations (0.98 to 125 μM) compared with the plot obtained with 2PAF (Fig. 1E). When we used these curves’ linear fit to calculate the LOD for NADH, we found that 2PPT is almost 20 times more sensitive to the NADH concentration than 2PAF (0.87 μM versus 17.5 μM). This value agrees well with the 2 to 4% quantum efficiency of NADH (*24*, *26*), considering that the rest of excitation photons mostly contribute to heat generation. Using a similar approach, we further verified our assumptions about the order-of-magnitude higher sensitivity of 2PPT compared to 2PAF in the case of FAD. Figure 1 (F and G) plots the 2PPT and 2PAF signals obtained for different concentrations of FAD solutions and the best linear fit. Also equivalent to the 8 to 10% quantum efficiency of FAD, we found that 2PPT gives an order-of-magnitude better sensitivity to the concentration of FAD when compared with 2PAF (0.99 μM versus 11.6 μM). While it shows lower sensitivity to FAD compared with NADH, which is expected on the basis of our assumption of lower autofluorescence emission/higher photothermal signal (and vice versa), 2PPT still exhibits LOD below the micromolar concentration that is better than fluorescence-based approaches. Note that the improved sensitivity of 2PAF to sense FAD (compared with NADH) is implicated by the tighter statistics it shows (Fig. 2E versus Fig. 2G). Overall, the high detection sensitivity of 2PPT to both NADH and FAD makes it a good candidate to study metabolism reprogramming in cancer cells and spheroids, where sensitive detection can image subtle alterations in the energetic balance after treatment.

### 2PPT microscopy senses metabolism coenzymes with enhanced specificity

In the next experiment, we characterize the imaging capability of 2PPT to dynamically report the concentration and localization of metabolic coenzymes and compare it with the widely used 2PAF modality. We use cultured SK-OV-3 cancer cells, grown in a 2D layer and freshly imaged within 45 min out of incubation at 37°C (see Materials and Methods). Figure 2 (A and D) illustrates the micrographs of cells from the same field of view (FoV) obtained using 2PPT and 2PAF, respectively. The excitation wavelength is set to λ_pump_ = 720 nm to target NADH molecule expression from subcellular organelles. For 2PAF detection, suitable emission filters were placed in front of the fluorescence detector according to the autofluorescence emission spectrum of the coenzyme (*41*). For more details about the 2PAF imaging setup, see Materials and Methods. An appreciable signal comes from cytoplasmic mitochondria, and the characteristic tubular-shaped mitochondria are observed along with the oval-shaped mitochondria. Figure 2B depicts a representative single cell from the imaged FoV, where both mitochondrial shapes can be observed clearly in the surrounding of the cell nucleus and some are marked with white (tubular) and yellow (oval) arrows. While the tubular, rod shape is widely associated with the mitochondrial structure, the oval, blob shape is also a common conformation of mitochondria that is associated with aging processes in cells, under-stress and diseased cells (*42*–*44*). Intensity histograms of the selected cellular region (blue bins) and background region (orange bins) are given in Fig. 2C, from which the signal-to-background ratio (SBR) of 14.27 dB for 2PPT was calculated on the basis of the means approach. Figure 2 (D to F) shows the 2PAF equivalents of Fig. 2 (A to C) and describes metrics obtained for the same cell FoV. Notably, 2PAF shows correspondence with the intense oval-shaped mitochondria observed in 2PPT; however, the NADH signal associated with the tubular mitochondria seems highly scattered, limiting the visibility of a single mitochondrion, which could arise from the lack of sensitivity for the specific biomolecule. A comparison of the intensity histograms for both modalities (Fig. 2C versus Fig. 2F) shows more than four times higher SBR for 2PPT compared with 2PAF. Given that the expression of cellular NADH is mainly associated with energy production and it mostly resides in cell mitochondria, it is crucial that as a cellular metabolism imaging system, 2PPT has the capability to delineate the difference between the two conformations of mitochondria. While elongated-shape mitochondria promote adenosine 5′-triphosphate production (ATP) and can be seen in healthy, energy-demanding cells, the oval/blob–shaped mitochondria are the fragmented version of the mitochondria commonly observed during cell stress or death processes (*45*). To validate the observed structure of mitochondria in the tested cell line, we labeled fresh cells with MitoTracker and obtained micrographs using 2P excited fluorescence imaging. Figure S3 illustrates different regions from the same dish of labeled cells, which confirms that both types of mitochondrial shapes exist in the tested cell line. Overall, the described tests and comparison show that 2PPT can detect NADH signal from a single mitochondrion, regardless of its shape, with better sensitivity than the equivalent 2PAF approach.

**Fig. 3.**
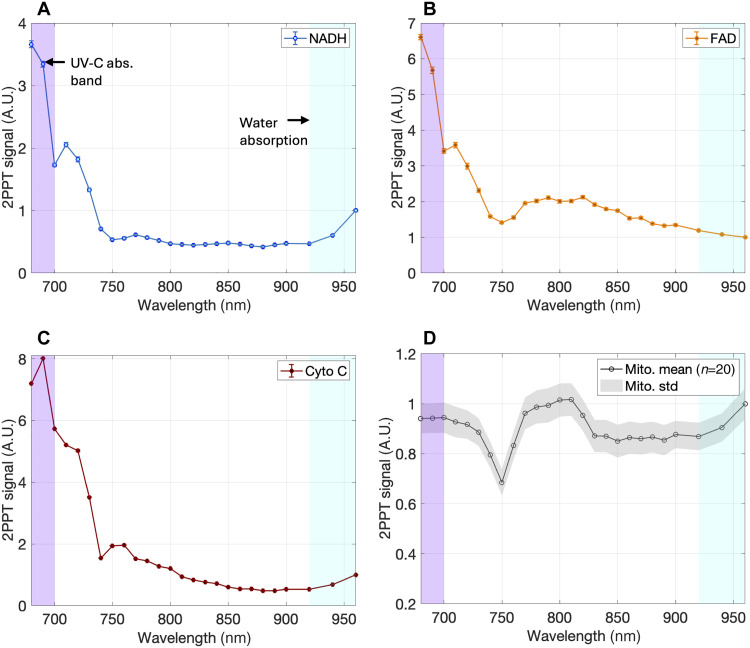
Validation of contrast source from mitochondria. 2PPT spectroscopy of (**A**) NADH, (**B**) FAD, (**C**) cytochrome C, and (**D**) mitochondria from live SK-OV-3 in the range of 680 to 960 nm. 2PPT spectroscopy of mitochondria shows similar spectroscopic features to the FAD coenzyme (dip around 750 nm and consistent signal between 770 and 820 nm). Bands associated with UV-C absorption and water absorption are highlighted by light purple and blue, respectively.

Unlike labeling optical microscopy approaches that use chemical probes to target the biomolecule of interest, 2PPT relies on local heat generation to provide the biomolecular contrast and thus lacks molecular specificity. Therefore, to further validate the contrast origin in 2PPT, we studied the spectral response of the observed subcellular features in the tested cells and compared it with pure sample standards. Other than metabolic coenzymes in living cells, heme proteins are considered major chromophores for 2P absorption in the NIR region that can potentially contribute to the local heat generation (*36*, *46*). Therefore, we studied the 2PPT spectral response of cytochrome C in addition to the metabolic coenzymes of interest, NADH and FAD. In this experiment, the pump beam, which dictates the 2P absorbance of the specific chromophore, was swept across the first NIR “biological window” relevant for subcellular features (*47*). Figure 3A plots the 2PPT signal of NADH solution (0.25 mM) as a function of pump beam wavelength. As expected, a strong 2PPT signal is observed around 720 nm that originates from the peak in 2P absorption of the chromophore (*41*, *42*) and the decrease thereafter. Notably, an even stronger signal is observed below 700 nm with a pronounced dip precisely at 700 nm. We attribute this behavior to the involvement of 2P excitation of the NADH biomolecules to the second excited state, which exhibit a very prominent one-photon (1P) absorption at the ultraviolet-C (UV-C) region (*42*). This higher, almost double, signal presumably originates from the combination of the UV-A tail (1P excitation at ~355 nm), which overlaps with the stronger UV-C contribution (1P excitation at ~260 nm) associated with transition to a higher electronic band (S_2_) (*48*). Figure 3B shows the spectral response for 0.25 mM FAD solution, where a relatively homogeneous 2PPT signal is observed over an extended bandwidth of 770 to 820 nm because of solid 2P absorption in this range. Given its prominent 1P absorption at UV-C wavelengths, the FAD spectrum also exhibits a strong signal below 700 nm, albeit with a less pronounced dip because of weak absorption around 720 nm (by contrast to NADH). The 2PPT spectral response of cytochrome C (0.25 mM solution), shown in Fig. 3C and obtained for a similar bandwidth to the NADH and FAD solutions, shows substantially different spectral features with a strong signal at 750 to 760 nm that steadily decays immediately afterward as it reaches 900 nm. To facilitate a quantitative comparison between the spectra shown in Fig. 3, we normalized each spectrum with respect to water absorption at 960 nm, given that each sample has the same solute concentration (*49*). Normalization of the entire spectrum by a scalar serves as a baseline correction to calibrate for uncontrolled distortions to the signal and is important to reliably capture the 2PPT spectrum relevant to our study, as shown in the case of NAD^+^ that has no 2PPT signal (fig. S4). The observed 2PPT spectroscopy of cytochrome C agrees with the strong single-photon absorption it has around 400 nm (*50*), by contrast to the weaker absorption by both the metabolic cofactors NADH and FAD in this region that is expressed by the low 2PPT signal around 750 nm. To determine the identity of the main contributor to heat generation when 2PPT is used in subcellular structures, we obtained spectral measurement of viable SK-OV-3 cells with different 2PPT pump wavelengths within the relevant window (see movie S1). Figure 3D shows the spectral response of 20 randomly selected mitochondria visible in the subcellular compartments. By inspecting the 2PPT spectra, one can appreciate the common spectroscopic features between the metabolic cofactors and the mitochondria, namely the declined signal (dip) around 750 nm and the relatively constant signal that starts at 770 nm and extends to 820 nm. These features are missing in the heme-protein spectrum. Notably, the strong signal below 700 nm that is observed in the solution samples is not observed in the cells. This is explained by decreased absorption from the adenine ring in the UV-C band, which occurs when the biomolecule is in bound state compared to its free state in aquatic samples (*51*). The similarity between these randomly selected organelles and the FAD spectral shape further suggests that under our experimental conditions, most of the signal associated with 2PPT originates from the metabolic coenzymes rather than the heme protein. To provide quantitative evaluation of this similarity, we compared the four spectra shown in Fig. 3 with phasor analysis, which compares their spectral shape regardless of intensity by projecting it to the complex phasor space (*52*). We only considered the 2PPT-relevant band of the spectrum between 700 and 900 nm (UV-C and water absorption excluded), and we found that the closest spectrum to that of mitochondria was FAD followed by NADH, while that of cytochrome C was the least similar (see fig. S5). Nevertheless, the subcellular contrast in photothermal imaging modalities is highly dependent on the experimental conditions (cell line, culture and preparation protocol, observation time, etc.) and is still an open debatable question. For example, it was previously shown that mitochondria are the major source of heat generation under 1P excitation in a different type of cell because of an ensemble of proteins (*46*).

### 2PPT imaging of metabolic perturbations in ovarian cancer cells

Metabolic perturbations are a hallmark of cancer, reflecting the profound reprogramming of cellular energy pathways that supports uncontrolled proliferation, survival under stress, and resistance to therapy (*53*). Investigating these perturbations under controlled conditions, such as nutrient deprivation and inhibition of reactive oxygen species (ROS), is essential for elucidating the adaptive mechanisms that enable cancer cells to survive within hostile microenvironments. The following experiments show the capability of 2PPT to image dynamic metabolic modifications in cells that stem from the biosynthesis of NADH and FAD during cancer cells’ growth and proliferation.

**Fig. 4.**
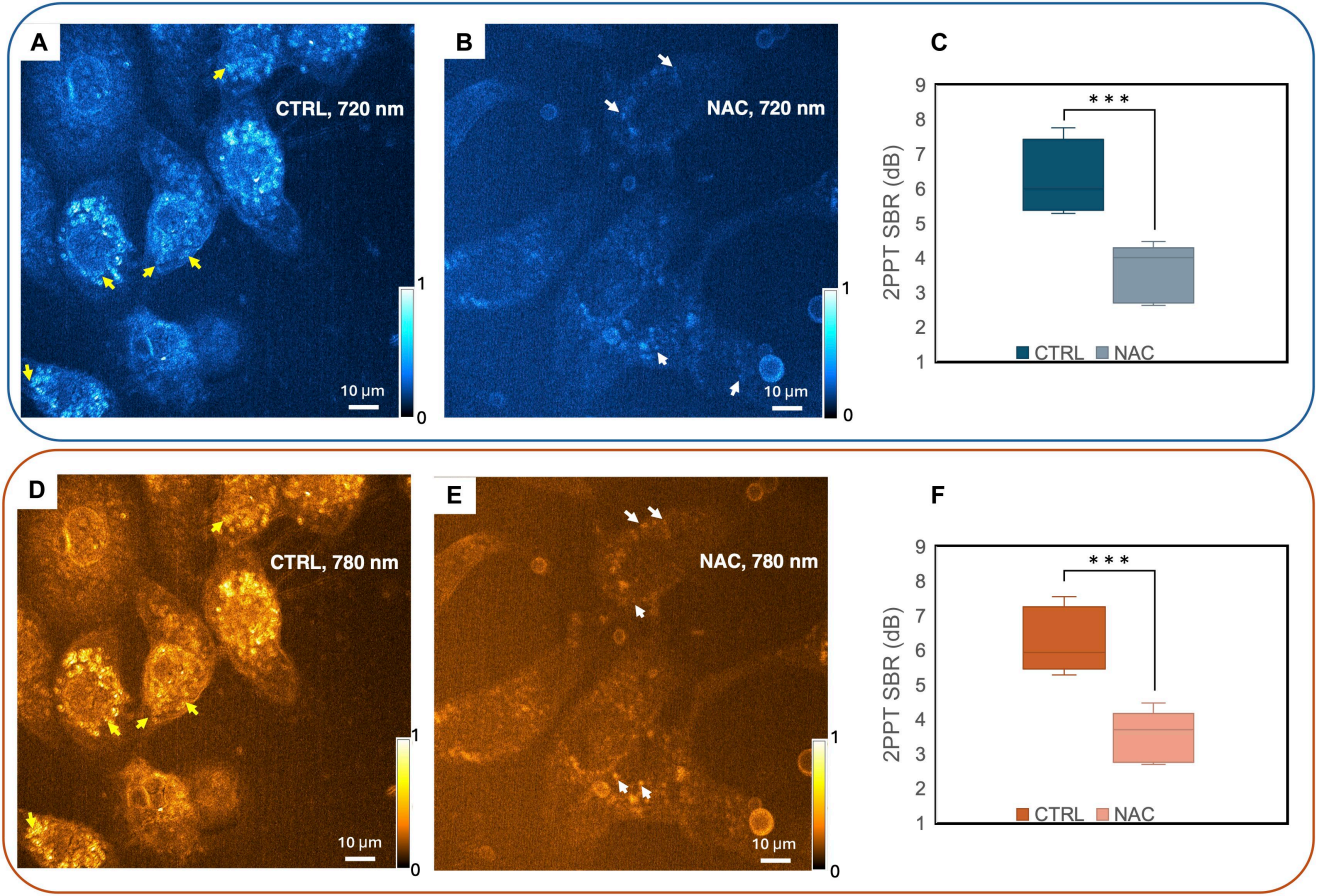
2PPT microscopy captures the metabolic response of SK-OV-3 cells to ROS inhibition. 2PPT micrographs obtained (**A**) without and (**B**) with NAC treatment, captured with a pump at 720 nm targeting NADH. Significant reduction in NADH coenzyme production is observed in (**C**). CTRL, untreated. (**D** and **E**) Equivalent images to (A) and (B) for the pump at 780 nm targeting FAD. Significant reduction in FAD coenzyme is observed in (**F**). Yellow and white arrows in micrographs mark regions of rod- and oval-shaped mitochondria, respectively. ***P < 0.0005.

We first studied the effects of ROS inhibition on the cells’ capacity to proliferate and produce energy. For this end, SK-OV-3 cells were cultured and grown as a single layer on a glass-bottom dish, as previously described. The treatment group consists of cells that were treated with *N*-acetylcysteine (NAC), which inhibits the production of ROS that are vital for energy production. Figure 4 (A and B) illustrates 2PPT micrographs of control and NAC-treated cells, respectively, obtained with a 720-nm pump beam (targets NADH). The box plot shown in Fig. 4C confirms the significantly reduced NADH levels in the NAC-treated cells compared to untreated control based on the measured SBR. Similarly, Fig. 4 (D to F) shows consistent trends derived from FAD expression in cells, obtained by tuning the pump beam to 780 nm. We observed significantly lower amounts of both coenzymes in the NAC-treated cells compared to control cells, with 33 and 37% reduction in NADH and FAD expression (median SBR value), respectively. Notably, the NAC-treated cells exhibit no mitochondria in the outer nuclear membrane compared to their existence in the untreated cells. Moreover, while the abundance of rod-shaped mitochondria is observed in the untreated cells (Fig. 4, A and D, yellow arrows), the ROS-inhibited cells show minimal (to not at all) rod-shaped microstructures in both channels and more oval-shaped micrometer-scale mitochondria within the cytoplasmic region (Fig. 4, B and E, white arrows). These observations of lower energy production accompanied by mitochondrial shape change might highlight an ongoing regularization process of the cells to compensate their dynamic energy needs (*54*, *55*).

**Fig. 5.**
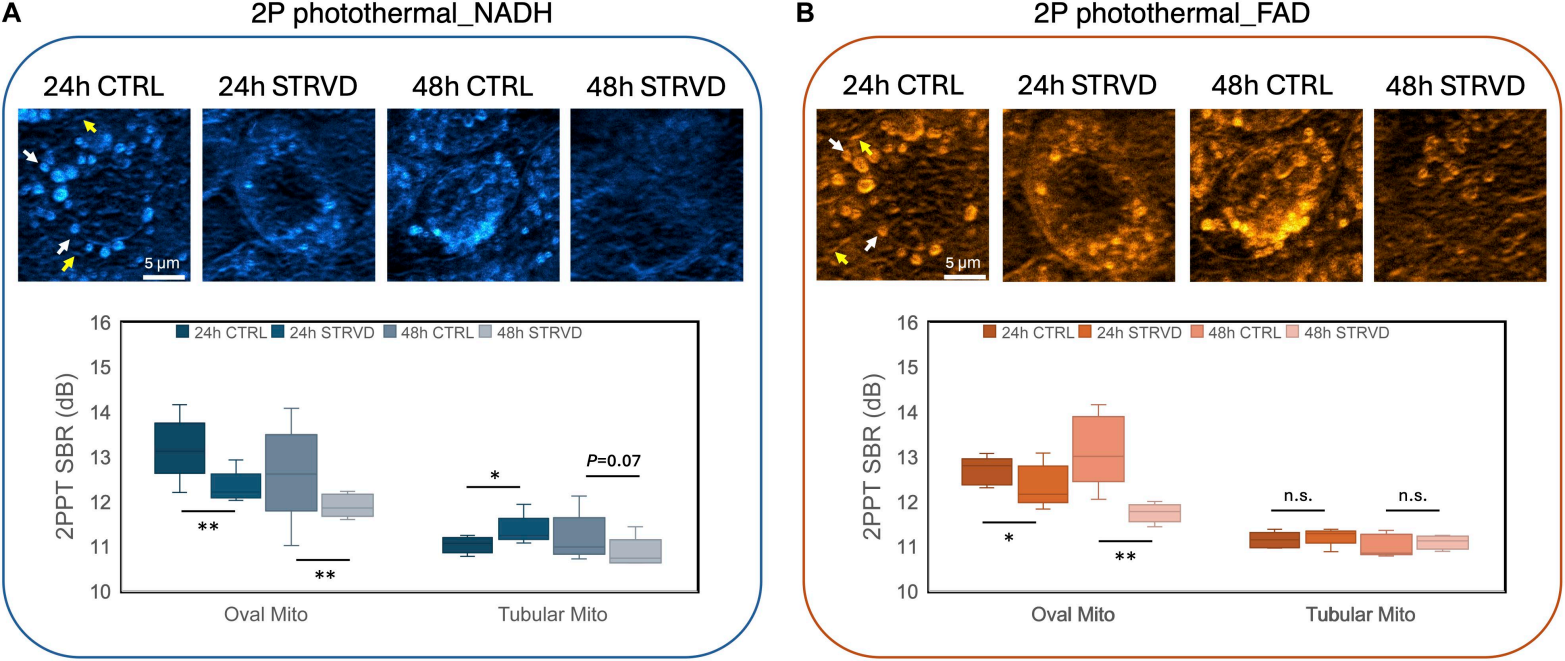
2PPT microscopy visualizes different mitochondrial morphologies in starved SK-OV-3 cells via NADH and FAD sensing. (**A**) Representative single-cell images (full field images in fig. S5) after 24- and 48-hour starvation along with their controls obtained from the NADH channel with the pump at 720 nm. The box plot below shows the trends observed for oval mitochondria (white arrows in 24-hour CTRL) and tubular mitochondria (yellow arrows in 24-hour CTRL). h, hour; STRVD, starved. (**B**) Equivalent data to (A) obtained from the FAD channel with the pump at 780 nm. n.s. is not significant, *P < 0.05, and **P < 0.005.

We further expanded our investigation of the metabolic reprogramming of cells under intentional disruption of energetic balance using 2PPT and incubated the cells to grow under starvation conditions for 24 and 48 hours (see Materials and Methods for more details). Compared with the equivalent control groups that were supplemented with sufficient nutrients during incubation, the starved cells activate protective responses, including autophagy to recycle internal components for energy and cell cycle arrest to reduce energy demands. Figure 5 summarizes the starvation experiment results for 24 and 48 hours as captured by both NADH and FAD channels in 2PPT. Data collected for NADH and FAD are grouped into separate panels (Fig. 5, A and B, respectively). Each panel contains a representative single-cell image from each condition in the experiment as labeled (24-hour control and starved and 48-hour control and starved). Few selected tubular mitochondria and oval mitochondria are marked in the left-most single-cell image by yellow and white arrows, respectively. Bar plots in Fig. 5 (A and B), below the single-cell frames, summarize our findings following the dual-time-point starvation experiment. To gain further insights into how these cells change their energy consumption and production, we divided the data obtained from *n* = 6 cells per condition (full FoV data in fig. S6) to the signal that comes from tubular (commonly healthy) mitochondria and oval-shaped (commonly unhealthy) mitochondria (*43*, *44*). To perform this task, we used image segmentation followed by shape and size analysis. Further details on how both types of mitochondria were identified and categorized can be found in Materials and Methods under the “Data analysis” section. Data collected from tubular mitochondria within the cells show a statistically significant increase in NADH (median SBR) levels following their starvation for 24 hours. Further starvation of these cells for 48 hours reveals a decline in NADH levels measured from tubular mitochondria. Although this difference did not reach statistical significance in our reported dataset (*P* = 0.07; Fig. 5A), the directionality of the change is consistent with a transient metabolic adaptation at 24 hours, potentially reflecting increased energetic allocation to maintain cellular processes, followed by a shift toward a more energy-conserving state at 48 hours. On the other hand, the NADH production from oval mitochondria follows a different trend with significant signal decay (median SBR) between the untreated and treated cells for both 24- and 48-hour starved cells. This suggests that the presumably unhealthy mitochondria are minorly associated with energy production that further declines when cells are under stress (starvation) conditions. Examination of the FAD related signal from the same location shows a nonsignificant change in the expression of the FAD levels (median SBR) from the tubular mitochondria. Meanwhile, the observed trends from oval mitochondria for FAD expression follow similar trends of NADH production, which might indicate that these dormant compartments are inactive during the cellular crisis and potentially have lost their functionality.

### 2PPT imaging of ovarian cancer spheroid response to chemotherapy

**Fig. 6.**
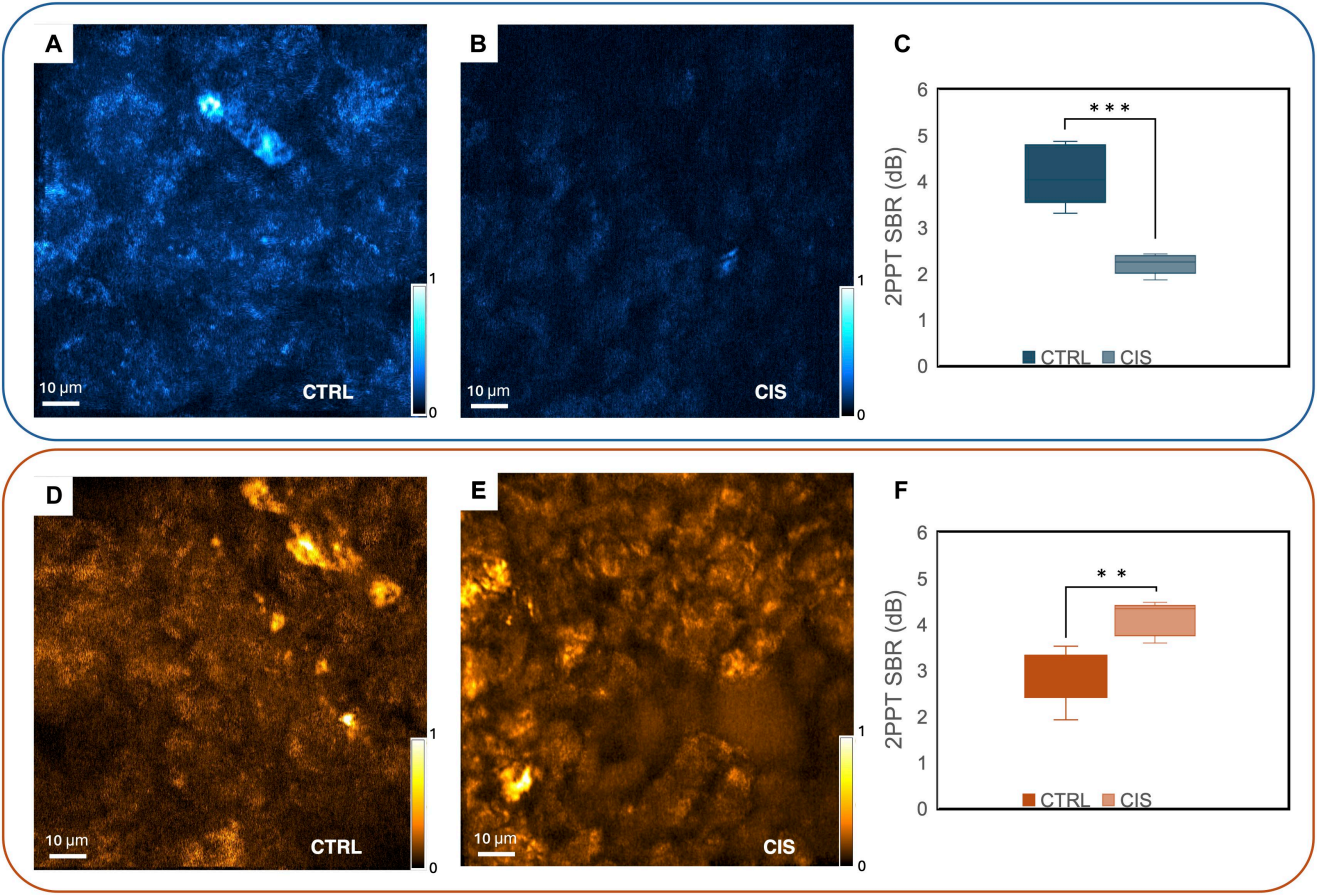
Metabolic heterogeneity in cancerous spheroids after chemotherapy treatment captured by 2PPT microscopy. MAPs of SK-OV-3 spheroids obtained with 2PPT for (**A**) untreated and (**B**) cisplatin-treated conditions captured with the pump at 720 nm targeting NADH. Significant reduction in coenzyme production is observed in (**C**). (**D** and **E**) Equivalent images to (A) and (B) for the pump at 780 nm targeting FAD. A significant increase in FAD coenzyme is observed in (**F**). CIS, cisplatin-treated. **P < 0.005, and ***P < 0.0005.

Tumor spheroids derived from ovarian cancer cells provide a physiologically relevant platform to investigate metabolic alterations under conditions that closely mimic the in vivo tumor microenvironment. Within these 3D constructs, gradients of oxygen, nutrients, and pH establish distinct metabolic niches that drive heterogeneity in cellular energy production and utilization (*56*–*58*). Characterizing these spatial metabolic alterations is therefore crucial for understanding how ovarian cancer cells adapt their mitochondrial function and redox balance to sustain proliferation, survive therapeutic stress, and develop resistance. NADH and FAD serve as intrinsic metabolic reporters whose optical signatures can reveal shifts in the balance between glycolytic and oxidative pathways across the spheroid architecture (*21*). Therefore, to investigate how cancer progression might be suppressed under chemotherapy treatment and assess the underlying mechanisms of tumor adaptations toward resistance, we study cancer spheroids under cisplatin drug treatment with the 2PPT microscope. Cultured SK-OV-3 ovarian cancer cells were supplemented in supporting tubes to facilitate their growth into spheroids of ~500 μm in diameter (see Materials and Methods for further details). Spheroids were then passaged and suspended in aquatic medium for imaging. Notably, 1P absorption of water from the third overtone in the ~720-nm band is one order of magnitude weaker than its second overtone in the ~970-nm band (*49*) and does not interfere with our 2PPT metabolic readouts, as demonstrated by the spectroscopic measurements of aquatic solutions and cells (Fig. 4 and movie S1). Maximum amplitude projections (MAPs) of 2PPT micrographs along the 100-μm-deep volume of untreated (labeled CTRL) and cisplatin-treated (labeled CIS) spheroids from both NADH and FAD channels (Fig. 6, A, B, D, and E), together with their respective 2PPT SBR statistics, are summarized in box plots (Fig. 6, C and F). From the depth-projected images of both NADH- and FAD-targeted photothermal maps, one can observe the nucleus membrane and cytoplasmic regions of individual cells constructing the tumor-mimicking spheroid. Inspection of the NADH map shows a sharp decline in intensity for the cisplatin-treated spheroid, while the equivalent FAD-associated map shows an opposite, albeit smaller, trend, with increased signal for the treated spheroid. These observations are further confirmed by comparing the 2PPT signal from the cytoplasmic regions of five cells per each spheroid. Statistically significant signal reduction of NADH is observed after the chemotherapy treatment (Fig. 6C), while a stronger signal is observed for FAD (Fig. 6F). Note that 2PPT SBR is reported as the signal levels in box plots (Fig. 6, C and F) to facilitate the comparison with other experimental data shown in this work (e.g., starvation test). The trends observed herein highlight the adaptability of cancerous cells to respond to an ongoing adversarial action from the hosting tumor. The different balance between the two metabolic coenzymes in untreated spheroids versus the treated ones may be a marker of ongoing metabolic adaptation within the tumor to facilitate its growth.

## DISCUSSION

It is well established that the pathogenesis and progression of carcinomas are linked to alterations in the metabolic activity of epithelial cells (*59*). The extensive tumor needs to supplement rapid growth, survival, and adaptation are met by substantial metabolic reprogramming of the cells, of which the Warburg effect, glutaminolysis, and lipogenesis are well-known hallmarks (*60*, *61*). These metabolic disorders have long been studied as predictive agents of oncogenic onset, given that they are associated with concomitant modification of the native cell environment at the (sub)microscale (*62*–*65*). The metabolic conditions of cells and tumors strictly influence the amount, distribution, and biochemical state of the endogenous chromophores within the biological system (*11*, *12*, *41*). Notably, NADH and FAD, which are almost the sole source for native fluorescence arising from the cell cytoplasm, were shown to carry important information about carcinogenesis and disease progression. The metastatic potential and disease stage are closely related to the microstructural and biochemical state of the precancerous cells as captured during the NADH and FAD consumption and production, rendering these coenzymes an important cancer biomarker (*3*, *66*). These results further fuel the ongoing efforts to target cellular metabolism to improve cancer therapeutics. The links between dysregulated cellular metabolism and cancer drug resistance hold great promise to enhance the efficacy of common therapeutic agents (*67*) and design combination therapies (*4*, *68*, *69*). Many of the mentioned findings were enabled by the perseverant fusion of interfacing areas of research commonly addressed to as biomedical imaging.

A key aspect of a successful microscopic technique for life science is the ability to extract biomedically relevant contrast in a label-free and nondestructive manner. Inspired by this notion, in this study, we demonstrate the potential of 2PPT microscopy as a sensitive and specific label-free imaging modality to probe metabolic-relevant biomolecules within living systems. By sensing the photothermal response of metabolic cofactors upon their 2P excitation, our approach surpasses conventional label-free fluorescence-based imaging in its ability to spatially map cellular metabolic activity with mitochondrial imaging. Traditional autofluorescence techniques that rely on photon emission from NADH and FAD often suffer from limited sensitivity because of background scattering, spectral overlap, and low quantum yields, constraining their accuracy in detecting subtle metabolic alterations. The experimental demonstrations presented herein establish 2PPT’s capability to report an enhanced signal-to-noise ratio and precise localization of mitochondrial metabolism, providing a versatile analytical window into dynamic redox states within living cells and 3D culture systems.

We first validated 2PPT sensitivity to endogenous metabolic biomolecules by demonstrating its submicromolar LOD, which is 10 and 20 times more sensitive than the well-established, widely used autofluorescence approach for FAD and NADH, respectively (*24*, *26*). It should be emphasized that the detection limits in both modalities are governed by different mechanisms (photothermal changes versus fluorescence emission). Therefore, the more efficient energy deposition in 2PPT compared to 2PAF is not the sole parameter that improves our detection capability, and the enhanced LOD in 2PPT is also attributed to the heat accumulation and thermal diffusion. This improvement in sensitivity has allowed us to visualize the heterogeneity of mitochondria compartments within cancer cells, highlighting the relationship between its structural variability and the cell redox state in a label-free manner (*54*, *55*). We identify spectroscopic measurement in 2PPT microscopy as a distinct feature that can provide chemical insights about the probed biological environment, decoupled from fluorescence emission inconsistencies. This was used in our studies to verify that the 2PPT signal from organelles mainly originates from metabolic cofactors (Fig. 2). The ability of our approach to detect perturbations in metabolism highlights its robustness and specificity in capturing a biochemical response. By intentionally disrupting the cellular metabolic activity (e.g., ROS inhibition and nutrient starvation), we demonstrate that 2PPT imaging accurately reflects intracellular alterations through distinct variations in the photothermal signatures of NADH and FAD. These findings establish a direct link between the optical contrast generated by our system and the functional metabolic response of the cell, confirming that our apparatus can serve as a quantitative, label-free probe of bioenergetic adaptation. This sensitivity to transient metabolic shifts positions 2PPT microscopy as a valuable complement to established biochemical assays, offering real-time, subcellular-level insight into metabolic regulation.

Imaging biomolecules from deep within 3D constructs that scramble light propagation is a long-standing effort in translational imaging (*70*, *71*). While we have not rigorously studied how the 2PPT signal scales compared to other fluorescence-based techniques in extraction of bulk-tissue interactions, we do recognize the potential it has to surpass current methodologies in achieving greater effective penetration. Spatial resolution in 2PPT is determined by the spatial overlap between the pump and probe beams. Meanwhile, the nonlinear nature of 2P excitation provides inherent optical sectioning and, therefore, 3D imaging capability. Given that an effective photothermal process can be induced with a non–diffraction-limited excitation beam, 2PPT can in principle be implemented with a probe beam that minimizes the difference in aberrations compared with the pump beam within the scattering medium and registers meaningful readouts deeper than fluorescently emitted photons. Overall, 2PPT, as a highly sensitive transient-based approach, can potentially register metabolically relevant information even in the case of severe distortion to its wavefront. Much like photoacoustic microscopy sensors (*72*), 2PPT has the capacity to sense relevant data as long as sufficient excitation is provided—and this is decoupled from the probe beam characteristics. Specifically, to leverage the 3D imaging capability of our approach, we extended our analysis to multicellular ovarian cancer spheroids to evaluate metabolic heterogeneity and treatment response. Within these physiologically relevant constructs, we observed a down-regulation of NADH expression concurrent with an up-regulation of FAD following chemotherapy exposure, reflecting a transition toward an oxidative metabolic phenotype. These results align with previous observations of redox remodeling in drug-treated cancer systems (*73*, *74*). The capacity to resolve such metabolic gradients across spheroidal volumes demonstrates the advantage of 2PPT over conventional fluorescence microscopy, which is often limited by scattering and photobleaching in dense biological samples.

Our current contribution follows a recent path of research that investigates approaches to develop label-free optical techniques for probing cellular metabolism and complementing and extending previously established modalities. Historically, autofluorescence imaging, particularly 2P excitation of NADH and FAD combined with fluorescence-lifetime measurements, has served as a foundational approach to map the redox state and metabolic dynamics in living cells and tissues (*3*, *7*, *11*, *12*, *14*, *41*). Photoacoustic imaging represents another powerful label-free route that achieves deep penetration and maps chromophores in thick tissues (*75*). More recently, multiphoton photoacoustic microscopy has been used to detect endogenous NADH deep in brain tissue (*76*). In addition, polarization ratiometric microscopy enables rapid, high-resolution tracking of NADH anisotropy as a functional readout of metabolic state without requiring lifetime measurements (*77*). These advances underscore a broader shift: combining absorption, thermal, acoustic, and polarization contrasts to report metabolism in more diverse, deeper, and more physiologically relevant contexts. Our 2PPT approach offers a distinct and complementary capability. By sensing photothermal signatures associated with NADH and FAD, we combine strong molecular sensitivity with multiphoton excitation’s spatial resolution and 3D imaging compatibility. This positions 2PPT as a valuable, label-free addition to emerging metabolic imaging platforms—bridging the gap between fluorescence-based lifetime methods, ultrafast absorption, and deep-tissue photoacoustic approaches.

While our study provides initial validation of 2PPT imaging for metabolic sensing, several aspects warrant further investigation, including optimization of detection parameters, calibration of photothermal signals with absolute metabolite concentrations, and extension to in vivo models. For instance, the direction and magnitude of NADH and FAD changes after therapy are sensitive to the drug mechanism, dose, and imaging time point—some reports show transient NADH increases (or decreases) depending on whether cells undergo early oxidative phosphorylation activation, oxidative stress, or apoptosis (*73*). Therefore, cross-study comparisons require careful matching of experimental conditions. Future studies should aim to integrate 2PPT with complementary imaging modalities and develop analytical models to correlate photothermal signatures with metabolic flux. Such efforts will be crucial for translating this methodology toward clinical and diagnostic applications in metabolic and oncologic research.

## MATERIALS AND METHODS

### Simulation of energy deposition and heat propagation to optimize 2PPT throughput

To evaluate the feasibility and enhance the registration of meaningful metabolic signal after optical heat deposition by the pump beam, we carried out a simulation to mimic the photothermally generated transients. Our simulation goals were to (i) verify thermal lensing, both spatially and temporally, within the affected volume and to (ii) estimate the needed relaxation time for sufficient heat buildup. These further helped us to optimize the needed pump and probe beam characteristics. Our chosen tested medium was 0.5 mM NADH solution. After calculating the amount of initial temperature rise deposited to the medium via *Q/*ρ*C*_p_, with *Q* being the total amount of absorbed energy, ρ being the molecule’s density, and *C*_p_ being the material’s heat capacity, our simulation started by solving the second-order partial differential equation known as the heat equation

$$\frac{\partial }{\partial t}T\left(r,t\right)=\alpha \nabla^{2}T\left(r,t\right)+\frac{Q}{\rho C_{p}}$$ (1)

where α is the thermal diffusivity, and *T* is the temperature. Equation 1 was solved for two cases during a single excitation-relaxation process: with a nonzero deposited energy term from the pump beam within the affected volume and with a zero deposited energy outside that volume. In accordance with the sample geometry (diameter >> thickness) and the raster scan acquisition of our system, to validate and optimize the system’s parameters, we used the following. The simulation was done on a 2D grid (*x*-*y*) mimicking transverse heat dissipation across the sample at the focal plane and used Dirichlet boundary conditions to account for the fixed surrounding temperature. For simplicity, we considered a flat-top pump beam of ~0.5 μm in diameter, which corresponds to the Airy disc of the used wavelength and optics, and a uniformly absorbed energy *Q*. Figure S1 describes the temperature field within the solution during the excitation of a single pulse and multiple pulses, from which an optimal number of photothermal events were calculated to set the experimental pump beam modulation (see subsequent section and the Supplementary Materials). In addition to time-resolved heat generation, fig. S1 shows a heatmap in different time points where a radially decaying temperature field is observed. Our simulation emphasizes that in our current 2PPT implementation, heat accumulation from multiple pulses is required to obtain a sufficient signal that is detectable by intensity measurements of the probe beam divergence (or convergence) through the thermal lens.

### 2PPT microscope

Our 2PPT microscope is based on acousto-optic–modulated (1205C-843, Isomet Corporation, Manassas, VA) pump beam (tunable, 680 to 960 nm) and an unmodulated probe beam [522.5-nm frequency doubled via a lithium triborate crystal (LBO-604H, EKSMA Optics, Vilnius, Lithuania), originally from a 1045-nm seed laser], both of which originate from an 80-MHz femtosecond solid-state ultrafast laser oscillator (InSight X3, Spectra-Physics, Andover, MA). To maximize the stability of the generated probe beam, the nonlinear crystal was illuminated with a focused beam and high peak power that was suppressed before interacting with the sample. Both beams collinearly directed to the back focal plane of a 60×, 1.2-NA water immersion microscope objective (Olympus, Japan) after they were steered by a pair of galvos (GVS002, Thorlabs, Newton, NJ) to raster-scan the sample of interest. On the sample, the probe beam size and probed volume thickness were ~350 nm and 10 μm, respectively. After interacting with the sample (dwell time, 20 μs), the probe beam signal was collected via a condenser lens equipped with an iris to dynamically sense photothermal lensing (*78*) and directed to a photodetector (DET100A2, Thorlabs, Newton, NJ) via a 525-nm bandpass optical filter (CT525/30, Chroma Technology Corp., Rockingham, VT). Average optical powers were set to 12 mW (pump beam) and 7 mW (probe beam), measured at the sample plane (no phototoxicity was observed). To enhance the signal, our photodetector was equipped with a 50-Ω resistor, a 22-kHz high-pass radio frequency filter, and a 46-dB low-noise amplifier (SA230-F5; Wayne). The photodetector (10-MHz bandwidth) output was sent to a lock-in amplifier (MFLI, Zurich Instruments USA Inc., Waltham, MA) that samples the signal at 60 MHz to satisfy the Nyquist criterion, and the 2PPT signal was recorded with a data acquisition card (NI-DAQ, PCI-6363, National Instruments, Austin, TX). The 2PPT system was further synchronized by the NI-DAQ card, which controlled the galvos scanning unit and the function generator that feed the AOM. To allow optimal energy deposition and heat generation, the function generator was set to rectangular wave modulation at 125 kHz and 5% duty cycle (no pulse shaping is involved). The amplified signal from the photodetector was demodulated by the pump beam reference and sent to a PC station to render and record the image.

### Preparation of NADH/FAD samples for characterization and LOD test

To characterize the 2PPT microscope, pure NADH and FAD samples were prepared in 1 mM stock solution from purchased high-purity cofactor powders (β-nicotinamide adenine dinucleotide, N8129; flavin adenine dinucleotide, F6625, MilliporeSigma, Burlington, MA) by mixing them with phosphate-buffered saline (PBS; pH 7.4, 1×, Gibco, Thermo Fisher Scientific, Waltham, MA) to achieve the desired concentration. For LOD tests, titration was made by halving the concentration of stock solution. To maintain a high accuracy of concentrations, a one-time 100 mM master solution was first made and then serially diluted to obtain the reported concentrations in the micromolar range. For each concentration in the LOD curve, a 1.3-μl solution was sandwiched between two 1.5# glass coverslips and mounted at the objective’s back focal plane. The sample thickness was controlled by a ~250-μm spacer used during the droplet encapsulation. The imaging medium was water, and the collection medium was oil. Data were obtained by imaging the droplet center with the 2PPT and 2PAF modalities, and the signal was extracted from a 5-μm-diameter centroid region (as shown in fig. S7). The signal was then plotted against the concentration, and the best linear fit was calculated using the MATALB R2023a curve fitting tool (MathWorks Inc., Natick, MA). For each condition, the residual standard deviation (σ) and the fit slope (*m*) were extracted, and the LOD was determined according to LOD = 3.3·σ/*m*, which is a standard approach for linear regression to calculate the value that represents when the signal is significantly different from the noise (*40*).

### Cell culture and spheroid preparation

SK-OV-3 cells (American Type Culture Collection) for both 2D and 3D cultures were cultured in the DMGM Basal Medium (Cell Applications, MCBD 105 Medium; Corning, Medium 199, 1×, combined 1:1) supplemented with 10% fetal bovine serum and penicillin/streptomycin (100 U/ml) and maintained in an incubator with 5% CO_2_ and 80 to 90% humidity at 37°C. For 2D cell culture samples, 24 hours before imaging, 75,000 to 100,000 cells were seeded in 35-mm glass-bottom dishes for control and treated groups in the complete DMGM medium. For treatment groups with NAC (Millipore Sigma-Aldrich, A91654), the cells were incubated with 5 mM NAC for 1.5 hours before imaging. For fluorescence imaging, the cells were incubated with MitoTracker (MitoTracker Green FM, M7514, Thermo Fisher Scientific, Waltham, MA) at a concentration of 100 nM for 15 min before imaging. For starvation experiments, the cells were seeded in the complete DMGM medium for 24 hours followed by 24- or 48-hour incubation in DMGM supplemented with 1 and 0.5% fetal bovine serum for different starvation levels. For live-cell imaging, the glass-bottom dishes with cells were washed once gently with 1× PBS, followed by immersion in 1× PBS. For 3D spheroid imaging, 8000 SK-OV-3 cells per well were seeded in ultralow-attachment round-bottom 96-well plates and incubated for 48 hours until the spheroids formed in the complete DMGM medium. For treatment groups with cisplatin (232120, MilliporeSigma, Burlington, MA), the spheroids were incubated at a final concentration of 10 μM for 4 hours. For live spheroid imaging, the spheroids were washed twice gently with 1× PBS and then transferred to a 35-mm glass-bottom dish with 1× PBS.

### 2PAF setup

To facilitate the comparison with 2PPT and ensure the localization of subcellular features between the two methods, 2PAF measurements were obtained using the 2PPT setup illustrated in Fig. 1A with the necessary modifications, as follows. The tunable port from our ultrafast laser was used as the light source for 2P excitation and was directed toward the sample using the same optics (AOM modulation off). To allow the collection of fluorescence signal in reflection mode, a dichroic mirror (DM; no. 69-899, Edmund Optics, Barrington, NJ) was introduced between the microscope objective and the galvos scanners. Emission was collected via the illumination objective with a 10-μs pixel dwell time and was directed toward a photomultiplier tube (H16722-40, Hamamatsu, Japan) after passing through a focusing lens and a chromatic filter to pick chromophore-specific signal 460/10 for NADH and 520/10 for FAD (FBH460-10 and FBH520-10, Thorlabs, Newton, NJ). The current signal from the photomultiplier tube was converted to measurable voltage by an in-house-built transimpedance amplifier equipped with 1-MHz low-pass filter and sent to the digitizer with no further operation. 2P excitation was verified by imaging 2.9-μm green-fluorescent beads (Fluoro-Max, G0300, Thermo Fisher Scientific) and confirming nonlinear scaling of the signal with the excitation power.

### Data analysis

A comparison of imaging performance between 2PPT and 2PAF modalities was done by selecting 25 patches (4 μm by 4 μm) from the cell-containing area and cell-free area in acquired micrographs (Fig. 2) to be accounted as the signal and background data, respectively. After manual classification of the micrograph regions to cell-containing (signal) and cell-free (noise), the patches from each class were randomly selected to avoid potential bias. We then analyzed the signal and noise histograms (Fig. 2), from which SBR was calculated in decibels on the basis of the means approach as 10·log(mean_signal_/mean_background_). Spectral responses of mitochondrial features to verify the source of contrast in 2PPT (Fig. 3D) were collected from the stack micrograph of cells obtained for the pump beam swept in the range of 680 to 960 nm (see movie S1). Twenty different regions (5 μm by 5 μm) containing different shapes of mitochondria were collected from six SK-OV-3 cells, and the means and standard deviation of 2PPT signal were used to plot the mitochondrial spectral response curve. Similarly, statistical data of 2PPT from subcellular mitochondria that were used in metabolic perturbation and chemotherapy assessment experimentation (Figs. 4 to 6) were obtained. A minimum of seven mitochondria-containing regions (5 μm by 5 μm) were registered per single cell, and data from a total of five or six cells were averaged per experimental condition (including control). To differentiate between rod- and oval-shaped mitochondria, we first discriminated the subcellular organelles from their background by applying a Gaussian smoothing filter, followed by an automated bimodal histogram–based image thresholding algorithm to maximize the separability of the resultant classes of noise and signal pixels (*79*). We then created a binary mask where mitochondria were defined as pixels above the automatically selected intensity threshold value. The different mask features were characterized on the basis of their shape, with a custom-built algorithm to identify elongated and oval structures in the image data. Last, the classified feature mask was merged in the signal extraction pipeline described above to report the signal values of the two mitochondrion types (Fig. 5). All these steps were specifically developed for this study and were implemented in MATLAB (R2023a, MathWorks Inc., Natick, MA). Executable code is available from the supplementary materials repository (see Data, code, and materials availability). To visualize spheroid data obtained from the 100-μm-thick volume of the interrogated constructs, we used the maximum intensity projection method. A custom-built MATLAB code to align the different slices separated by ~20 μm was used to account for lateral distortion and sampling errors between the registered planes, followed by projecting the maximum value per lateral location within the volume onto the 2D micrographs to obtain the MAPs (Fig. 6).

### Statistical analysis

The statistical graphs were shown as the means ± standard deviation, unless specified otherwise. The statistical analysis was done using either an unpaired *t* test between treated and untreated groups. Statistical significance was denoted as *P < 0.05, **P < 0.005, ***P < 0.0005, and n.s. for *P* > 0.05, indicating a nonsignificant statistical difference.

## Supplementary Materials

### The PDF file includes:

Supplementary Text Figs. S1 to S7 Legend for movie S1 References
