# Supplementary material for: Submicromolar imaging of intrinsic chromophores by two-photon photothermal microscopy captures mitochondrial response to chemotherapy
Source: Sci Adv. 2026 Jul 10;12(28):eaee7678. doi: 10.1126/sciadv.aee7678 (PMC13353361; doi:10.1126/sciadv.aee7678)
Supplement: Supplementary file 1 — Supplementary Text Figs. S1 to S7 Legend for movie S1 References [file sciadv.aee7678_sm.pdf]

Science Advances
AAAS

Supplementary Materials for

Submicromolar imaging of intrinsic chromophores by two-photon photothermal microscopy captures mitochondrial response to chemotherapy

Nathaniel Hai *et al.*

Corresponding author: Ji-Xin Cheng, jxcheng@bu.edu

Sci. Adv. **12**, eaee7678 (2026)
DOI: 10.1126/sciadv.aee7678

The PDF file includes:

Supplementary Text
Figs. S1 to S7
Legend for movie S1
References

Other Supplementary Material for this manuscript includes the following:

Movie S1

Supplementary text

Equivalence between signal generation process in 2PPT and 2PAF

Here we revisit the contrast mechanism in both modalities to emphasize the expected equivalence between generation of autofluorescence signal and photothermal signal that are measured during two-photon excitation of chromophore biomolecule. With reference to Figure 1(C) in main text, it is important to note that by equivalence here we mean that the signal ratio between any two measurements of a chromophore with both methods would roughly be similar. Let us consider that 2PPT generated signal is the highest temperature rise after one cycle of two-photon absorption events, then the signal commensurate with $I_{2PPT} \sim (I_{2p}^2 \cdot M \cdot \eta_{abs})/(\rho \cdot C_p)$, with M being the chromophore's molar concentration, I_{2p} the two-photon excitation peak power, η_{abs} the chromophore two-photon absorption cross section, and $\rho \cdot C_p$ represents the volumetric heat capacity of the surrounding medium (80,81). Similarly, we will neglect scattering effects in cells/tissues and consider that autofluorescence generation follows $I_{2PAF} \sim I_{2p}^2 \cdot M \cdot \eta_{abs}$ (82). This gives a basis as to why 2PPT is more efficient than 2PAF in sensing the metabolic biomoleucles as demonstrated by our LOD results (approximately 20X better in NADH and 10X better in FAD, refering to **Figure 1**). By inspecting I_{2PPT} and I_{2PAF}, the improved performance mainly stems from extraction of the signal and efficiency of the relaxation processes after absorption, both of which diminish the signal levels in 2PAF and favor the 2PPT readout. Additionally, because both 2PPT and 2PAF scale with the two-photon absorption cross section and chromophore concentration, the expected 2PPT signals across different biomolecular solutions and excitation wavelengths predict proportional behavior relative to 2PAF. More specifically, we obtained I_{2PPT}(NADH; 720 nm)/ I_{2PPT}(NADH; 780 nm)=11.35 and I_{2PPT}(FAD; 720 nm)/I_{2PPT}(FAD; 780 nm)=2.13. For comparison, data retrieved from (40) for action cross section gives σ_{2p}(NADH;720 nm)/σ_{2p} (NADH;780 nm)=11.75 and σ_{2p}(FAD;720 nm)/σ_{2p} (FAD;780 nm)=2.11. We see a very good match, which together with higher LOD of 2PPT for both NADH and FAD [**Figure 1(D)-Figure 1(F)**] highlights the better sensitivity and specificity of 2PPT to native chromophores.

Calculation of limit of detection (LOD)

Here we show the calculation of LOD of the sterile samples measured, NADH and FAD, with reference to Figures 1(D)-1(G). As we mentioned in the main text, the LOD for both of the approaches, 2PPT and 2PAF, is calculated based on the linear regression curve and the formula LOD=$3.3 \cdot \sigma / m$, with σ being the residuals standard deviation and m the slope of the regression line. Below are the extracted values and the LOD calculation for each condition in the order they appear in the main text, as follows:

- 2PPT_LOD_NADH = 3.3·1.8E-3/6.8E-3 = 0.87 μM
- 2PAF_LOD_NADH = 3.3·6.9E-3/1.3E-3 = 17.5 μM
- 2PAF_LOD_NADH = 3.3·1.8E-3/6.0E-3 = 0.99 μM
- 2PAF_LOD_FAD = 3.3·2.1E-3/0.6E-3 = 11.6 μM

Note that LOD units are determined by the horizontal axis units with σ in signal units (A.U.) and m acts as a conversion factor in units of signal/concentration (A.U./μM).

Figs. S1 to S7

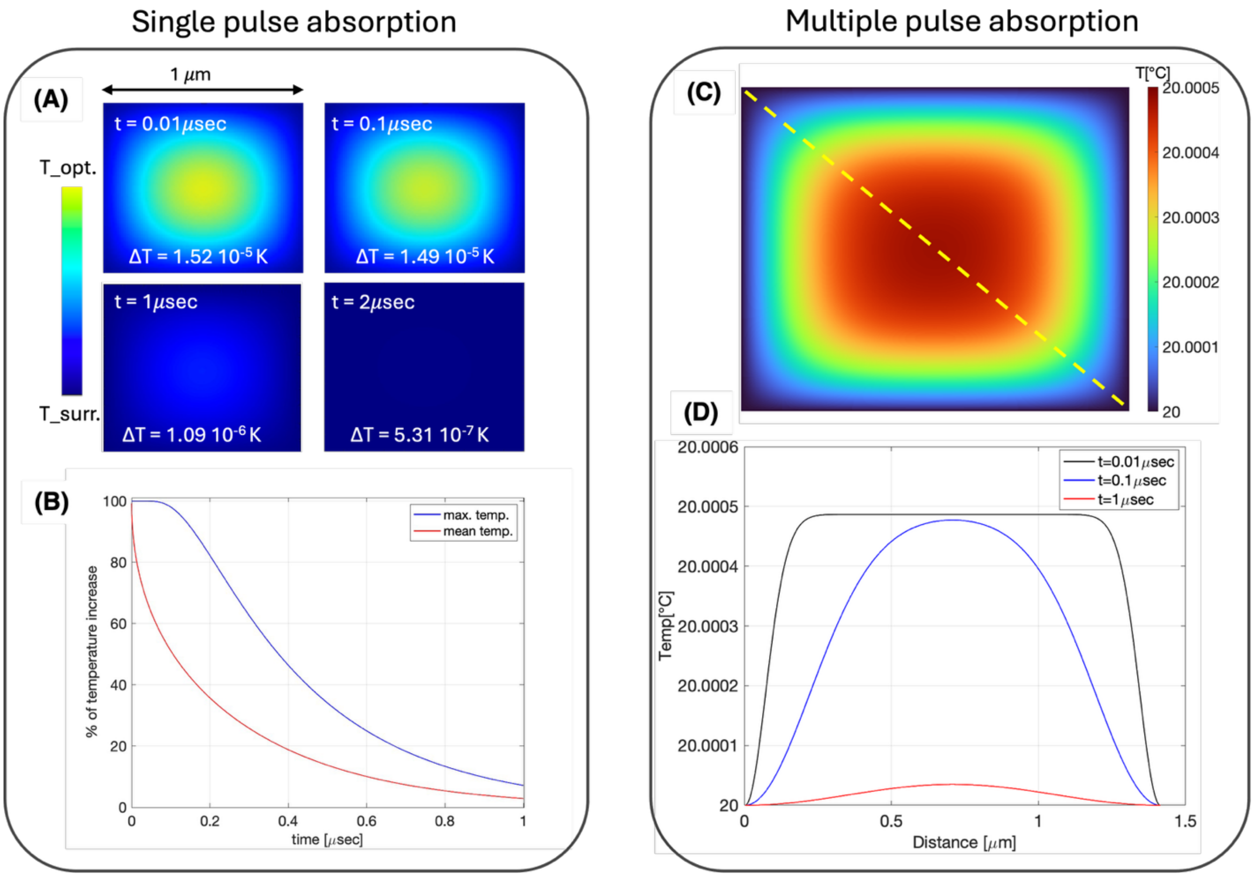

Fig. S1. Simulation results of 2PPT signal in NADH solution after single pulse and multiple pulse absorption. (A) Temperature map of a 1 μm x 1 μm square around the pump beam focus at different times post the single absorption event (top of each frame). The temperature rise at the center of impacted region $\Delta T(t)=T_{opt}(t)-T_{surr}$ is indicated in the bottom of each frame. **(B)** Plots of maximum and mean temperature increase compared to the surrounding temperature (i.e., outside of the impacted area by the pump beam focus) $100\cdot\Delta T(t)/\Delta T(t=0^+)$ as a function of time after the absorption event (i.e., at $t=0^+$ sec). Left panel shows that temperature rise after a single absorption is negligible and heat is not accumulated to a detectable level. **(C)** Temperature map of the 1 μm x 1 μm square around the 12 mW (average) pump beam focus 0.1 μs after 32 photothermal absorption events, where a detectable increase in temp is reached (~0.5 mK rise). **(D)** Profile of temperature map along the dashed Yellow line in **(C)** showing that temperature almost decays back to the surrounding temperature level 1 μs after photothermal absorption events. Simulation results verify that, with the used 80 MHz laser at 12 mW average power, a 125 KHz pulse train modulation with 5% duty cycle (corresponds to 400 ns pulse train of 32 pulses) produces sufficiently detectable heat.

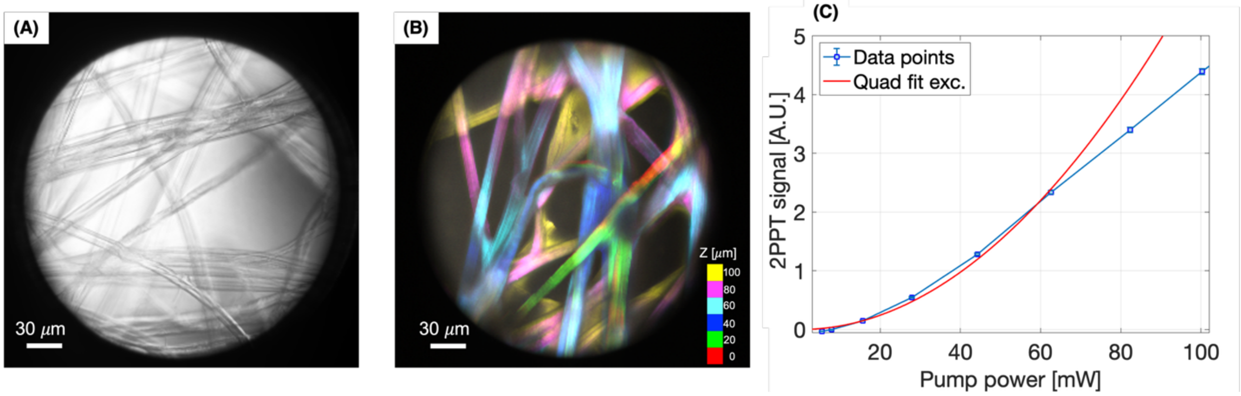

Fig. S2. Validation of 2-photon excitation in 2PPT microscope. (A) Brightfield and **(B)** 2PPT images of a non-woven web of elongated strands observed in a lens-cleaning tissue. Tissue was soaked with a 0.25 mM FAD solution and dried for 2h to render strands as photothermally active media. Brightfield image captured from a single plane shows in focus and out of focus strands. Depth color coded 2PPT image consists of 20 µm equally spaced planes within the 100 µm thickness tissue. Comparison shows the confocal rejection characteristic of multiphoton excitation (83). **(C)** 2PPT signal obtained from a 0.25mM FAD solution as a function of pump power intensity (blue) and the corresponding quadratic fit (red) according to $I_{2PPT} = a \cdot I_{2p}^2 + b$. Quadratic fit excludes two highest power data points (pump power equals 83 mW and 100 mW) to better reflect the nonlinear dependence of generated heat on the pump power. Deviation from quadratically generated signal at high laser peak powers is due to depletion of ground state molecules to be excited to a higher electronic state (84).

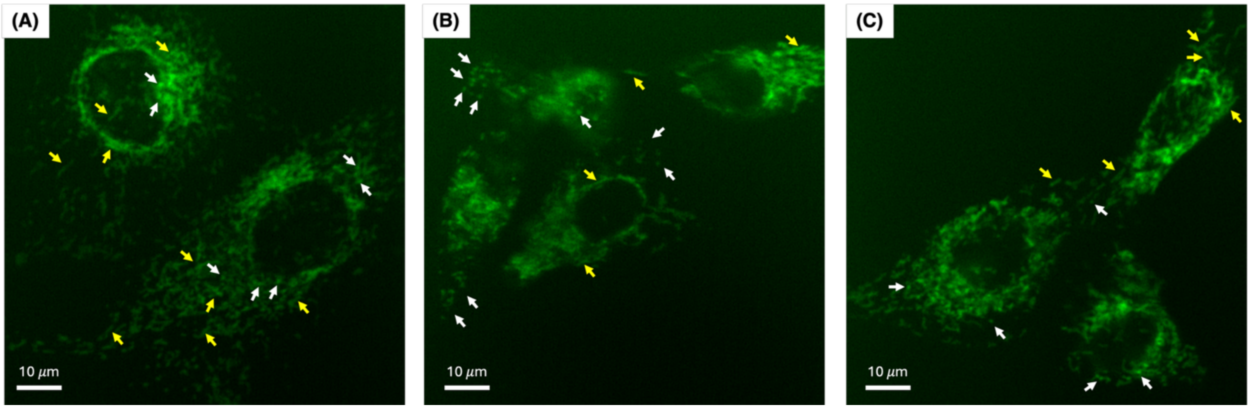

Fig. S3. Verifying the various mitochondria conformations by 2 photon fluorescence of SK-OV-3 cells labeled with Mito tracker. Different fields of view obtained from similarly treated cells showing that tubular- and oval-shaped mitochondria coexist in the cancerous cell line studied in this work. Yellow and white arrows point to tubular and oval mitochondria, respectively.

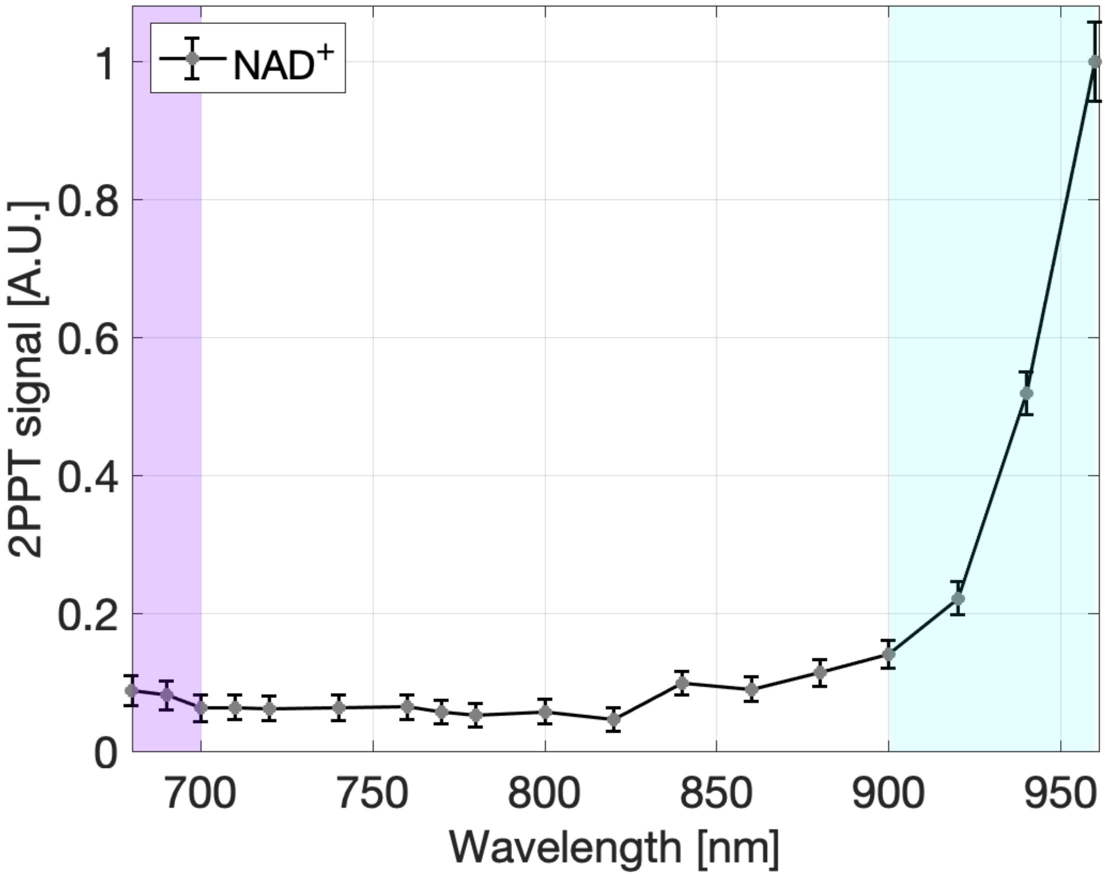

Fig. S4. 2PPT spectroscopy of pure NAD^+ solution. Sample concentration is 0.25 mM and signal was obtained under same condition as spectra from Figure 3 with similar normalization with respect to water absorption in 960 nm. With no normalization the baseline level is ~0.7. Water absorption and 1P UV-C bands are highlighted by light blue and purple, respectively.

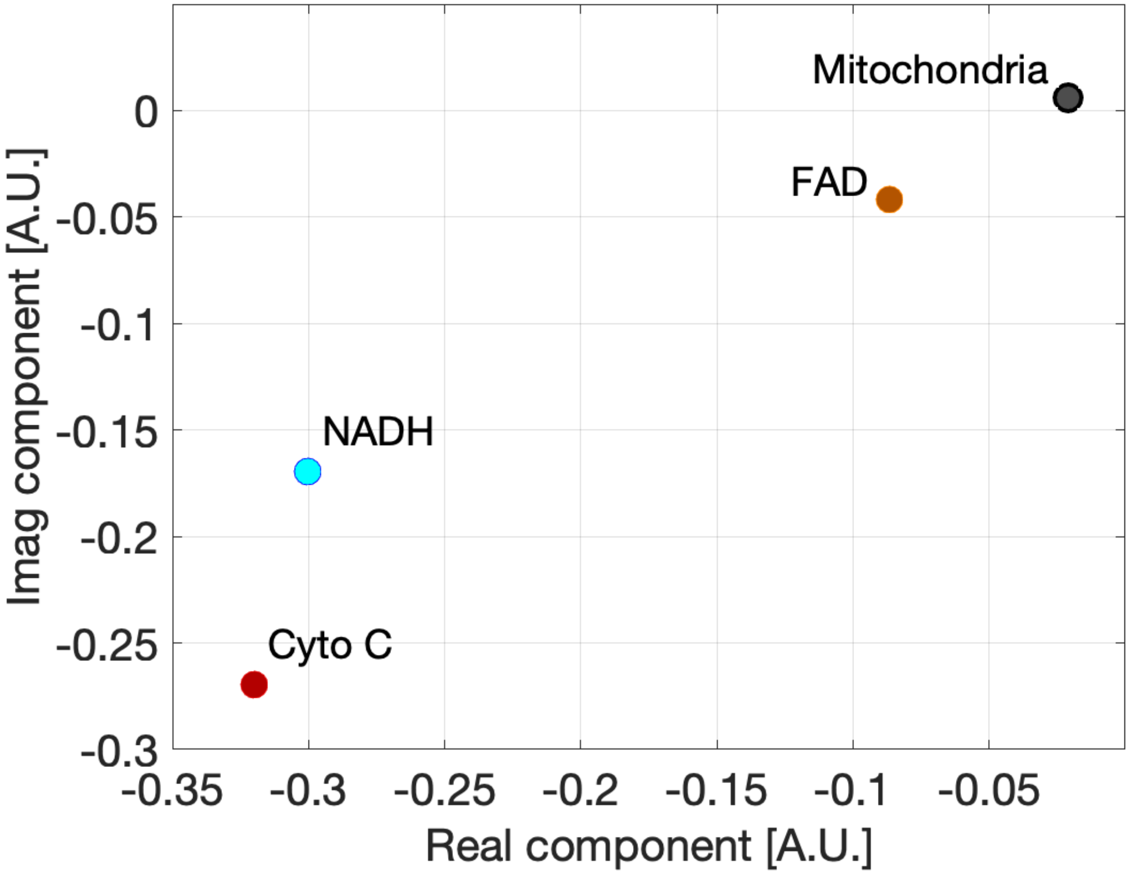

Fig. S5. Phasor analysis of 2PPT spectral response showing most of heat in mitochondria comes from metabolic coenzymes. Each data point represents the projection of 2PPT spectrum (biological window, 700-900 nm) to the complex phasor space. Euclidian distance is used to determine that FAD spectrum has the highest resemblance to the mitochondria spectrum, followed by NADH. Cytochrome C spectrum is the least similar to mitochondria. This dataset confirms that, under our experimental conditions, 2PPT signal in cells mainly comes from the activity of metabolic coenzymes rather the hemeproteins.

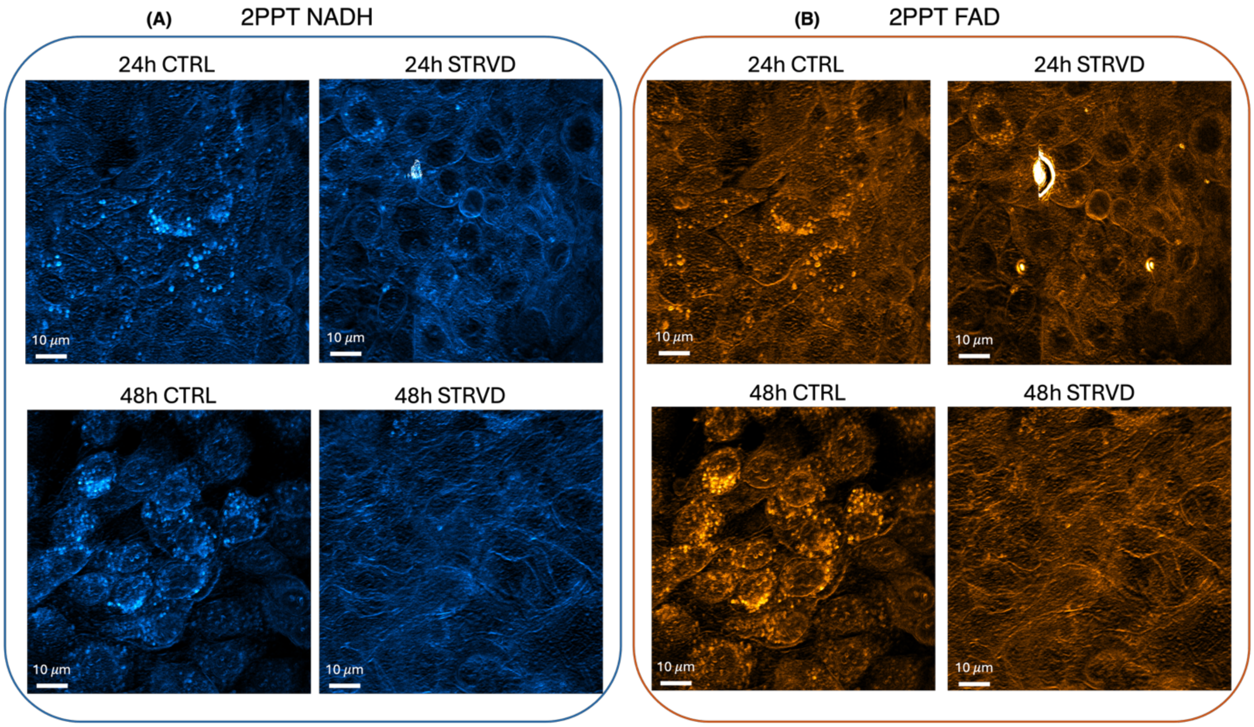

Fig. S6. Full field images of SK-OV-3 cells in starvation conditions and controls. (A) Shows frames from which NADH data was acquired and **(B)** are equivalent frames for FAD data. As labeled on top, frames for 24h and 48h starvation cells are shown along with their controls of nutrition-supplemented cells.

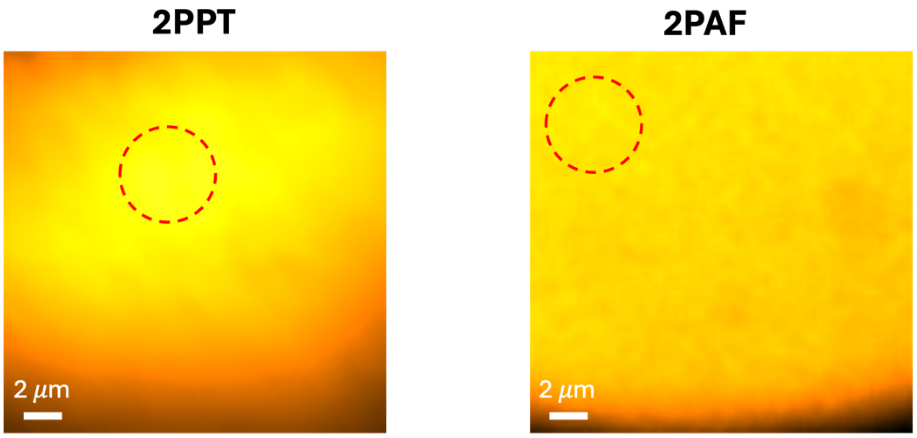

Fig. S7. Example of signal extraction from droplet images used to evaluate LOD curves. Dashed red circle shows a 5 μm diameter centroid region of 125 μM FAD droplet imaged with **(A)** 2PPT and **(B)** 2PAF.

Movie caption

Movie S1. Validation of contrast source from mitochondria in 2PPT. Micrographs of unlabeled SK-OV-3 cells obtained at different pump wavelengths of 680 nm – 960 nm in 2PPT, from which spectral shape of mitochondria was obtained [**Figure 3(D)**]. Annotated frames that show the regions from which mitochondria signal was obtained are shown on the right panel (labeled with red squares).

REFERENCES

1. S. Huang, A. A. Heikal, W. W. Webb, Two-photon fluorescence spectroscopy and microscopy of NAD (P) H and flavoprotein. *Biophys. J.* **82**, 2811–2825 (2002).

2. L. Z. Li, H. N. Xu, M. Ranji, S. Nioka, B. Chance, Mitochondrial redox imaging for cancer diagnostic and therapeutic studies. *J. Innov. Opt. Health Sci.* **2**, 325–341 (2009).

3. M. C. Skala, K. M. Riching, A. Gendron-Fitzpatrick, J. Eickhoff, K. W. Eliceiri, J. G. White, N. Ramanujam, In vivo multiphoton microscopy of NADH and FAD redox states, fluorescence lifetimes, and cellular morphology in precancerous epithelia. *Proc. Natl. Acad. Sci. U.S.A.* **104**, 19494–19499 (2007).

4. T. Nakamura, M. Conrad, Exploiting ferroptosis vulnerabilities in cancer. *Nat. Cell Biol.* **26**, 1407–1419 (2024).

5. H. N. Xu, J. Tchou, M. Feng, H. Zhao, L. Z. Li, Optical redox imaging indices discriminate human breast cancer from normal tissues. *J. Biomed. Opt.* **21**, 114003–114003 (2016).

6. Z. Liu, D. Pouli, C. A. Alonzo, A. Varone, S. Karaliota, K. P. Quinn, K. Münger, K. P. Karalis, I. Georgakoudi, Mapping metabolic changes by noninvasive, multiparametric, high-resolution imaging using endogenous contrast. *Sci. Adv.* **4**, 9302 (2018).

7. S. You, H. Tu, E. J. Chaney, Y. Sun, Y. Zhao, A. J. Bower, Y. Z. Liu, M. Marjanovic, S. Sinha, Y. Pu, S. A. Boppart, Intravital imaging by simultaneous label-free autofluorescence-multiharmonic microscopy. *Nat. Commun.* **9**, 2125 (2018).

8. P. F. Favreau, J. He, D. A. Gil, D. A. Deming, J. Huisken, M. C. Skala, Label-free redox imaging of patient-derived organoids using selective plane illumination microscopy. *Biomed. Opt. Express* **11**, 2591–2606 (2020).

9. I. Georgakoudi, M. C. Skala, K. P. Quinn, C. Stringari, J. E. Sorrells, A. A. Heikal, L. Z. Li, H. N. Xu, S. You, A. J. Walsh, R. Datta, K. Samimi, A. A. Gillette, K. W. Eliceiri, M. Balu, S. A. Boppart, M. A. Digman, K. R. Dunning, C. L. Evans, A. A. Garcia, J. P. Houston, W. Hwang, M. M. Lindley, X. Li, Z. Liu, L. Marcu, S. Murugkar, M. G. Nichols, R. Niesner, S. H. Parekh,

N. Rajaram, S. Ranjit, K. Shen, L. Shi, B. Torrado, A. Vallmitjana, M. Wagner-Evers, R. Zemp, Consensus guidelines for cellular label-free optical metabolic imaging: Ensuring accuracy and reproducibility in metabolic profiling. *J. Biomed. Opt.* **30**, S23901–S23901 (2025).

10. P. Luu, S. E. Fraser, F. Schneider, More than double the fun with two-photon excitation microscopy. *Commun. Biol.* **7**, 364 (2024).

11. A. Varone, J. Xylas, K. P. Quinn, D. Pouli, G. Sridharan, M. E. McLaughlin-Drubin, C. Alonzo, K. Lee, K. Münger, I. Georgakoudi, Endogenous two-photon fluorescence imaging elucidates metabolic changes related to enhanced glycolysis and glutamine consumption in precancerous epithelial tissues. *Cancer Res.* **74**, 3067–3075 (2014).

12. A. J. Walsh, R. S. Cook, H. C. Manning, D. J. Hicks, A. Lafontant, C. L. Arteaga, M. C. Skala, Optical metabolic imaging identifies glycolytic levels, subtypes, and early-treatment response in breast cancer. *Cancer Res.* **73**, 6164–6174 (2013).

13. I. Miranda-Lorenzo, J. Dorado, E. Lonardo, S. Alcala, A. G. Serrano, J. Clausell-Tormos, M. Cioffi, D. Megias, S. Zagorac, A. Balic, M. Hidalgo, M. Erkan, J. Kleeff, A. Scarpa, B. Sainz Jr., C. Heeschen, Intracellular autofluorescence: A biomarker for epithelial cancer stem cells. *Nat. Methods* **11**, 1161–1169 (2014).

14. I. Georgakoudi, K. P. Quinn, Label-free optical metabolic imaging in cells and tissues. *Annu. Rev. Biomed. Eng.* **25**, 413–443 (2023).

15. L. Marcu, Fluorescence lifetime techniques in medical applications. *Ann. Biomed. Eng.* **40**, 304–331 (2012).

16. Y. Sun, S. You, H. Tu, D. R. Spillman Jr., E. J. Chaney, M. Marjanovic, J. Li, R. Barkalifa, J. Wang, A. M. Higham, N. N. Luckey, K. A. Cradock, Z. G. Liu, S. A. Boppart, Intraoperative visualization of the tumor microenvironment and quantification of extracellular vesicles by label-free nonlinear imaging. *Sci. Adv.* **4**, eaau5603 (2018).

17. S. You, R. Barkalifa, E. J. Chaney, H. Tu, J. Park, J. E. Sorrells, Y. Sun, Y.-Z. Liu, L. Yang, D. Z. Chen, M. Marjanovic, S. Sinha, S. A. Boppart, Label-free visualization and characterization of extracellular vesicles in breast cancer. *Proc. Natl. Acad. Sci. U.S.A.* **116**, 24012–24018 (2019).

18. D. Entenberg, M. H. Oktay, J. S. Condeelis, Intravital imaging to study cancer progression and metastasis. *Nat. Rev. Cancer* **23**, 25–42 (2023).

19. T. M. Heaster, A. J. Walsh, Y. Zhao, S. W. Hiebert, M. C. Skala, Autofluorescence imaging identifies tumor cell-cycle status on a single-cell level. *J. Biophotonics* **11**, e201600276 (2018).

20. J. N. Stoecker, S. Udgata, A. A. Gillette, C. A. Pasch, M. C. Skala, D. A. Deming, Using patient-derived cancer organoids to determine the effects of the anti-EGFR therapy panitumumab in the presence or absence of KRAS mutation. *Cancer Res.* **84**, 1944–1944 (2024).

21. A. Gillette, S. Udgata, A. E. Schmitz, J. N. Stoecker, J. D. Kratz, D. A. Deming, M. C. Skala, Wide-field optical redox imaging with leading-edge detection enables assessment of treatment response and heterogeneity in patient-derived cancer organoids. *Cancer Res.* **85**, 4329–4340 (2025).

22. J. E. Sorrells, J. Park, E. Aksamitiene, M. Marjanovic, E. M. Martin, E. J. Chaney, A. M. Higham, K. A. Cradock, Z. G. Liu, S. A. Boppart, Label-free nonlinear optical signatures of extracellular vesicles in liquid and tissue biopsies of human breast cancer. *Sci. Rep.* **14**, 5528 (2024).

23. C. Xu, W. R. Zipfel, Multiphoton excitation of fluorescent probes. *Cold Spring Harb. Protoc.* **2015**, 086116 (2015).

24. T. M. Cannon, J. L. Lagarto, B. T. Dyer, E. Garcia, D. J. Kelly, N. S. Peters, A. R. Lyon, P. M. W. French, C. Dunsby, Characterization of NADH fluorescence properties under one-photon excitation with respect to temperature, pH, and binding to lactate dehydrogenase. *OSA Contin.* **4**, 1610–1625 (2021).

25. T. Itoh, Fluorescence and phosphorescence from higher excited states of organic molecules. *Chem. Rev.* **112**, 4541–4568 (2012).

26. I. A. Gorbunova, M. K. Danilova, M. E. Sasin, V. P. Belik, D. P. Golyshev, O. S. Vasyutinskii, Determination of fluorescence quantum yields and decay times of NADH and FAD in water–alcohol mixtures: Analysis of radiative and nonradiative relaxation pathways. *J. Photochem. Photobiol. A* **436**, 114388 (2023).

27. G. Weng, X. Zhao, J. Zhao, J. Li, J. Zhu, J. Zhao, Nanoplasmonic sensing of NADH by inhibiting the oxidative etching of gold nanorods. *Sens. Actuators B Chem.* **299**, 126982 (2019).

28. P. Jain, B. Chakma, S. Patra, S. Goswami, Hairpin stabilized fluorescent silver nanoclusters for quantitative detection of NAD^+ and monitoring NAD^+/NADH-based enzymatic reactions. *Anal. Chim. Acta* **956**, 48–56 (2017).

29. J. Chen, J. Wang, Y. Geng, J. Yue, W. Shi, C. Liang, W. Xu, S. Xu, Single-cell oxidative stress events revealed by a renewable SERS nanotip. *ACS Sens.* **6**, 1663–1670 (2021).

30. W. Wang, S. Ruan, Z. Su, P. Xu, Y. Chen, Z. Lin, J. Chen, Y. Lu, A novel "on–off" SERS nanoprobe based on sulfonated cellulose nanofiber–Ag composite for selective determination of NADH in human serum. *Microchim. Acta* **190**, 254 (2023).

31. S. Cao, Z. Zhou, H. Li, M. Jia, Y. Liu, M. Wang, M. Zhang, S. Zhang, J. Chen, J. Xu, J. R. Knutson, A fraction of NADH in solution is "dark": Implications for metabolic sensing via fluorescence lifetime. *Chem. Phys. Lett.* **726**, 18–21 (2019).

32. A. Gaiduk, M. Yorulmaz, P. V. Ruijgrok, M. Orrit, Room-temperature detection of a single molecule's absorption by photothermal contrast. *Science* **330**, 353–356 (2010).

33. M. E. Durst, J. Mertz, "Multiphoton photothermal imaging in scattering samples" in *Optics in the Life Sciences* (Optica Publishing Group, 2011).

34. J. Moger, N. L. Garrett, D. Begley, L. Mihoreanu, A. Lalatsa, M. V. Lozano, M. Mazza, A. Schatzlein, I. Uchegbu, Imaging cortical vasculature with stimulated Raman scattering and two-photon photothermal lensing microscopy. *J. Raman Spectrosc.* **43**, 668–674 (2012).

35. M. M. Bijeesh, P. K. Shakhi, S. Arunkarthick, G. K. Varier, P. Nandakumar, Confocal imaging of single $BaTiO_3$ nanoparticles by two-photon photothermal microscopy. *Sci. Rep.* **7**, 1643 (2017).

36. S. Lu, W. Min, S. Chong, G. R. Holtom, X. S. Xie, Label-free imaging of heme proteins with two-photon excited photothermal lens microscopy. *Appl. Phys. Lett.* **96**, 113701 (2010).

37. A. Gaiduk, P. V. Ruijgrok, M. Yorulmaz, M. Orrit, Detection limits in photothermal microscopy. *Chem. Sci.* **1**, 343–350 (2010).

38. Q. Xia, Y. Jiaze, G. Zhongyue, J.-X. Cheng, Mid-infrared photothermal microscopy: Principle, instrumentation, and applications. *J. Phys. Chem. B* **126**, 8597–8613 (2022).

39. W. R. Zipfel, R. M. Williams, R. Christie, A. Y. Nikitin, B. T. Hyman, W. W. Webb, Live tissue intrinsic emission microscopy using multiphoton-excited native fluorescence and second harmonic generation. *Proc. Natl. Acad. Sci. U.S.A.* **100**, 7075–7080 (2003).

40. B. Altshuler, B. Pasternack, Statistical measures of the lower limit of detection of a radioactivity counter. *Health Phys.* **9**, 293–298 (1963).

41. A. C. Croce, G. Bottiroli, Autofluorescence spectroscopy and imaging: A tool for biomedical research and diagnosis. *Eur. J. Histochem.* **58**, 2461 (2014).

42. P. M. Schaefer, S. Kalinina, A. Rueck, C. A. von Arnim, B. von Einem, NADH autofluorescence—A marker on its way to boost bioenergetic research. *Cytometry A* **95**, 34–46 (2019).

43. T. Ahmad, K. Aggarwal, B. Pattnaik, S. Mukherjee, T. Sethi, B. K. Tiwari, M. Kumar, A. Micheal, U. Mabalirajan, B. Ghosh, S. S. Roy, Computational classification of mitochondrial shapes reflects stress and redox state. *Cell Death Dis.* **4**, e461 (2013).

44. Y. Zhou, Q. Long, H. Wu, W. Li, J. Qi, Y. Wu, G. Xiang, H. Tang, L. Yang, K. Chen, L. Li, F. Bao, H. Li, Y. Wang, M. Li, X. Liu, Topology-dependent, bifurcated mitochondrial quality control under starvation. *Autophagy* **16**, 562–574 (2020).

45. B. C. Jenkins, K. Neikirk, P. Katti, S. M. Claypool, A. Kirabo, M. R. McReynolds, A. Hinton, Mitochondria in disease: Changes in shapes and dynamics. *Trends Biochem. Sci.* **49**, 346–360 (2024).

46. D. Lasne, G. A. Blab, F. D. Giorgi, F. Ichas, B. Lounis, L. Cognet, Label-free optical imaging of mitochondria in live cells. *Opt. Express* **15**, 14184–14193 (2007).

47. G. Hong, A. Antaris, H. Dai, Near-infrared fluorophores for biomedical imaging. *Nat. Biomed. Eng.* **1**, 0010 (2017).

48. J. Kim, S. H. Lee, F. Tieves, C. E. Paul, F. Hollmann, C. B. Park, Nicotinamide adenine dinucleotide as a photocatalyst. *Sci. Adv.* **5**, eaax0501 (2019).

49. J. E. Bertie, Z. Lan, Infrared intensities of liquids XX: The intensity of the OH stretching band of liquid water revisited, and the best current values of the optical constants of H_2O(l) at 25°C between 15,000 and 1 cm^{-1}. *Appl. Spectrosc.* **50**, 1047–1057 (1996).

50. M. Hulko, I. Hospach, N. Krasteva, G. Nelles, Cytochrome C biosensor—A model for gas sensing. *Sensors* **11**, 5968–5980 (2011).

51. A. Cadena-Caicedo, B. Gonzalez-Cano, R. López-Arteaga, N. Esturau-Escofet, J. Peon, Ultrafast fluorescence signals from β-dihydronicotinamide adenine dinucleotide: Resonant energy transfer in the folded and unfolded forms. *J. Phys. Chem. B* **124**, 519–530 (2019).

52. F. E. Robles, J. W. Wilson, M. C. Fischer, W. S. Warren, Phasor analysis for nonlinear pump-probe microscopy. *Opt. Express* **20**, 17082–17092 (2012).

53. N. N. Pavlova, J. Zhu, C. B. Thompson, The hallmarks of cancer metabolism: Still emerging. *Cell Metab.* **34**, 355–377 (2022).

54. X. Liu, G. Hajnóczky, Altered fusion dynamics underlie unique morphological changes in mitochondria during hypoxia–reoxygenation stress. *Cell Death Differ.* **18**, 1561–1572 (2011).

55. Y. Miyazono, S. Hirashima, N. Ishihara, J. Kusukawa, K. I. Nakamura, K. Ohta, Uncoupled mitochondria quickly shorten along their long axis to form indented spheroids in a fission-independent manner. *Sci. Rep.* **8**, 350 (2018).

56. K. Ganesh, C. Wu, K. P. O'Rourke, B. C. Szeglin, Y. Zheng, C.-E. G. Sauvé, M. Adileh, I. Wasserman, M. R. Marco, A. S. Kim, M. Shady, A rectal cancer organoid platform to study individual responses to chemoradiation. *Nat. Med.* **25**, 1607–1614 (2019).

57. S. N. Bess, G. K. Smart, M. J. Igoe, T. J. Muldoon, Scaffold-free development of multicellular tumor spheroids with spatial characterization of structure and metabolic radial profiles. *In Vitro Models* **3**, 91–108 (2024).

58. S. Riffle, R. S. Hegde, Modeling tumor cell adaptations to hypoxia in multicellular tumor spheroids. *J. Exp. Clin. Cancer Res.* **36**, 102 (2017).

59. G. A. Wagnieres, W. M. Star, B. C. Wilson, In vivo fluorescence spectroscopy and imaging for oncological applications. *Photochem. Photobiol.* **68**, 603–632 (1998).

60. M. Tufail, C. H. Jiang, N. Li, Altered metabolism in cancer: Insights into energy pathways and therapeutic targets. *Mol. Cancer* **23**, 203 (2024).

61. J. Li, S. Condello, J. Thomes-Pepin, X. Ma, Y. Xia, T. D. Hurley, D. Matei, J. X. Cheng, Lipid desaturation is a metabolic marker and therapeutic target of ovarian cancer stem cells. *Cell Stem Cell* **20**, 303–314 (2017).

62. D. K. Bird, L. Yan, K. M. Vrotsos, K. W. Eliceiri, E. M. Vaughan, P. J. Keely, J. G. White, N. Ramanujam, Metabolic mapping of MCF10A human breast cells via multiphoton fluorescence lifetime imaging of the coenzyme NADH. *Cancer Res.* **65**, 8766–8773 (2005).

63. A. A. Heikal, Intracellular coenzymes as natural biomarkers for metabolic activities and mitochondrial anomalies. *Biomark. Med.* **4**, 241–263 (2010).

64. T. Chang, M. S. Zimmerley, K. P. Quinn, I. Lamarre-Jouenne, D. L. Kaplan, E. Beaurepaire, I. Georgakoudi, Non-invasive monitoring of cell metabolism and lipid production in 3D engineered human adipose tissues using label-free multiphoton microscopy. *Biomaterials* **34**, 8607–8616 (2013).

65. K. Drozdowicz-Tomsia, A. G. Anwer, M. A. Cahill, K. N. Madlum, A. M. Maki, M. S. Baker, E. M. Goldys, Multiphoton fluorescence lifetime imaging microscopy reveals free-to-bound NADH ratio changes associated with metabolic inhibition. *J. Biomed. Opt.* **19**, 086016 (2014).

66. K. Alhallak, L. G. Rebello, T. J. Muldoon, K. P. Quinn, N. Rajaram, Optical redox ratio identifies metastatic potential-dependent changes in breast cancer cell metabolism. *Biomed. Opt. Express* **7**, 4364–4374 (2016).

67. Y. Zhao, E. B. Butler, M. J. C. D. Tan, Targeting cellular metabolism to improve cancer therapeutics. *Cell Death Dis.* **4**, e532 (2013).

68. A. Podsednik, J. Jiang, A. Jacob, L. Z. Li, H. N. Xu, Optical redox imaging of treatment responses to Nampt inhibition and combination therapy in triple-negative breast cancer cells. *Int. J. Mol. Sci.* **22**, 5563 (2021).

69. N. Santana-Codina, A. S. Chandhoke, Q. Yu, B. Małachowska, M. Kuljanin, A. Gikandi, J. M. Stańczak, S. Gableske, M. P. Jedrychowski, D. A. Scott, A. J. Aguirre, W. Fendler, N. S. Gray, D. Mancias, Defining and targeting adaptations to oncogenic KRASG12C inhibition using quantitative temporal proteomics. *Cell Rep.* **30**, 4584–4599 (2020).

70. F. Helmchen, W. Denk, Deep tissue two-photon microscopy. *Nat. Methods* **2**, 932–940 (2005).

71. S. Yoon, S. Y. Cheon, S. Park, D. Lee, Y. Lee, S. Han, M. Kim, H. Koo, Recent advances in optical imaging through deep tissue: Imaging probes and techniques. *Biomater. Res.* **26**, 57 (2022).

72. V. Ntziachristos, Going deeper than microscopy: The optical imaging frontier in biology. *Nat. Methods* **7**, 603–614 (2010).

73. A. T. Shah, T. M. Heaster, M. C. Skala, Metabolic imaging of head and neck cancer organoids. *PLOS ONE* **12**, e0170415 (2017).

74. M. Broekgaarden, A. L. Bulin, J. Frederick, Z. Mai, T. Hasan, Tracking photodynamic- and chemotherapy-induced redox-state perturbations in 3D culture models of pancreatic cancer. *J. Clin. Med.* **8**, 1399 (2019).

75. B. Dong, H. Li, Z. Zhang, K. Zhang, S. Chen, C. Sun, H. F. Zhang, Isometric multimodal photoacoustic microscopy based on optically transparent micro-ring ultrasonic detection. *Optica* **2**, 169–176 (2015).

76. T. Osaki, W. D. Lee, X. Zhang, R. E. Zubajlo, M. Balcells-Camps, E. R. Edelman, B. W. Anthony, M. Sur, P. T. So, Multi-photon, label-free photoacoustic and optical imaging of NADH in brain cells. *Light Sci. Appl.* **14**, 264 (2025).

77. L. Ling, J. C. Crowley, M. L. Tan, J. A. M. R. Kunitake, A. A. Shimpi, R. M. Williams, L. A. Estroff, C. Fischbach, W. R. Zipfel, Assessing cellular metabolic dynamics with NAD(P)H fluorescence polarization imaging. bioRxiv 667273 (2025). www.biorxiv.org/content/10.1101/2025.07.28.667273v2.

78. Y. Zhu, X. Ge, H. Ni, J. Yin, H. Lin, L. Wang, Y. Tan, C. V. P. Dessai, Y. Li, X. Teng, J. X. Cheng, Stimulated Raman photothermal microscopy toward ultrasensitive chemical imaging. *Sci. Adv.* **9**, eadi2181 (2023).

79. N. Otsu, A threshold selection method from gray-level histograms. *Automatica* **11**, 285–296 (1979).

80. O. Blum, N. T. Shaked, Prediction of photothermal phase signatures from arbitrary plasmonic nanoparticles and experimental verification. *Light Sci. Appl.* **4**, e322 (2015).

81. Y. Ren, Y. Yan, H. Qi, Photothermal conversion and transfer in photothermal therapy: From macroscale to nanoscale. *Adv. Colloid Interface Sci.* **308**, 102753 (2022).

82. J. R. Albani, *Principles and Applications of Fluorescence Spectroscopy* (Wiley-Blackwell, Oxford, 2007).

83. W. R. Zipfel, R. M. Williams, W. W. Webb, Nonlinear magic: Multiphoton microscopy in the biosciences. *Nat. Biotechnol.* **21**, 1369–1377 (2003).

84. W. Denk, J. H. Strickler, W. W. Webb, Two-photon laser scanning fluorescence microscopy. *Science* **248**, 73–76 (1990).
